# Detection of Hidden Maize Weevil Infestation in Wheat Under Varying Moisture Conditions Using an Asymmetric Dual-Stream Masked Autoencoder Fusion Network

**DOI:** 10.3390/foods15214000

**Published:** 2026-11-07

**Authors:** Honglin Ma, Zibo Zhu, Lei Yan, Taoying Luo, Chao Zhao, Chunqi Bai, Xiaoyu Wu

**Affiliations:** 1 College of Information Science and Engineering, Henan University of Technology, Zhengzhou 450001, China; zhuzb@stu.haut.edu.cn (Z.Z.); wuxiaoyu@stu.haut.edu.cn (X.W.); 2 School of Food and Strategic Reserves, Henan University of Technology, Zhengzhou 450001, China; yanlei@haut.edu.cn (L.Y.); 2018050@haut.edu.cn (C.Z.); baigrain@haut.edu.cn (C.B.); 3 Guangzhou Grain Group Co., Ltd. Reserve Branch, Guangzhou 510000, China; luotaoying06@163.com

**Keywords:** maize weevil, hidden infestation, varying moisture conditions, near-infrared hyperspectral imaging, masked autoencoder

## Abstract

Early detection of hidden maize weevil (*Sitophilus zeamais*) infestation in wheat is difficult, and moisture variation can obscure weak near-infrared spectral responses. This study investigated wheat in three nominal moisture groups designated as 13%, 16% and 19%. Hyperspectral data and corresponding three-channel pseudocolor images were acquired from healthy and infested kernels at four operationally defined sampling times to construct single- and mixed-moisture datasets. An Asymmetric Dual-Stream Masked Autoencoder Fusion Network (ADS-MAFNet) was developed by combining a CNN–Transformer spectral branch with masked autoencoder (MAE) pretraining and a 2D-CNN image branch for binary classification. Classification performance varied across the nominal moisture groups, with most spectral deep learning models performing less well in the 19% group. Within the investigated datasets, ADS-MAFNet achieved higher mean accuracy and F1-score than the evaluated single-branch neural models in the nominal 19% and mixed-moisture groups. Its mixed-moisture accuracy, precision, recall, and F1-score were 79.40%, 75.27%, 84.28%, and 79.44%, respectively. However, PLS-DA achieved higher mean accuracy and F1-score than ADS-MAFNet under all four evaluated moisture conditions. Ablation comparisons showed larger ten-run mean differences for branch removal than for removal of MAE pretraining, but a consistent advantage over IMG-only and a consistent pretraining benefit were not established. Classification performance was generally higher at the two later sampling times than at the two earlier sampling times. These results support further investigation of hyperspectral image fusion within the represented laboratory conditions; transfer to unseen moisture levels and independent batches remains untested.

## 1. Introduction

Wheat is susceptible to hidden infestation by storage pests such as the maize weevil (*Sitophilus zeamais*). Maize weevil larvae feed and develop inside the kernels, while external symptoms remain inconspicuous during the early stages of infestation. Consequently, early infestation is difficult to identify by manual inspection and can reduce wheat quality and economic value [1,2]. Therefore, developing a rapid and nondestructive method for detecting early internal pest infestation in wheat kernels is important for pest monitoring and quality assurance during storage.

Near-infrared spectroscopy and hyperspectral imaging have been used to detect internal insect infestation and damaged wheat kernels [3,4]. Hyperspectral imaging has subsequently been applied to detecting hidden rice weevil infestation [5] and discriminating insect-damaged or otherwise damaged wheat kernels [6,7]. However, wheat moisture content varies after harvest, and the spectral differences associated with early hidden maize weevil infestation are inherently weak. Under varying moisture conditions, infestation-related signals can be masked by the moisture background. In practical storage scenarios, differences in moisture content directly affect spectral reflectance and absorption characteristics [8,9,10], thereby complicating detection.

Under varying moisture conditions, spectral features alone may therefore be insufficient for stable discrimination between healthy and infested samples. Hyperspectral (HSI) data can capture spectral responses associated with changes in kernel composition and internal structure, whereas the corresponding three-channel pseudocolor images (IMG) retain spatial information at selected wavelengths that may complement the mean spectra. Previous work combining near-infrared hyperspectral data with digital images for insect-damaged wheat kernels has demonstrated the complementary value of these information sources in pest detection [11]. Accordingly, this study examined whether combining mean spectra and pseudocolor images improved classification within the investigated nominal moisture groups.

Early hidden maize weevil infestation produces only subtle differences between healthy and infested kernels, while sample acquisition is constrained by the insect developmental cycle and the need for consistent developmental stages, increasing the difficulty of model training. Deep learning can extract discriminative features from high-dimensional hyperspectral data [12]. Masked autoencoders (MAEs) learn internal data structure by masking and reconstructing portions of the input [13]. In hyperspectral analysis, MAE-based approaches have been applied to spectral reconstruction and hyperspectral image classification, providing a methodological basis for MAE pretraining of the hyperspectral branch in this study [14,15].

In summary, hyperspectral imaging has shown considerable potential for detecting hidden insect infestation in wheat. However, the relative performance of conventional spectral classifiers and spectral image neural models across different moisture backgrounds remains insufficiently characterized for hidden infestation detection. Because near-infrared spectral responses are highly sensitive to moisture, changes in wheat moisture may obscure weak infestation-related information and reduce the stability of hyperspectral classification. Therefore, this study investigated wheat in three nominal moisture groups designated as 13%, 16%, and 19% and constructed both single-moisture and mixed-moisture datasets using paired hyperspectral data and corresponding pseudocolor images. An asymmetric dual-stream model was developed to combine an MAE-pretrained CNN–Transformer spectral encoder with a lightweight image encoder operating on pseudocolor crops from the same hyperspectral acquisition. The contribution of this study lies in jointly examining representation fusion, spectral pretraining, and a conventional spectral comparator across the investigated nominal moisture groups and operational sampling times. The comparisons characterize the benefits and limitations of fusion and spectral pretraining relative to neural and conventional spectral baselines. The framework provides an empirical assessment of these design choices, not evidence of established moisture invariance or superiority of deep learning. The study aimed to compare classification performance across the investigated nominal moisture groups, assess the contribution of spectral image fusion, and analyze detection differences across nominal developmental sampling times. The mixed-moisture evaluation included moisture groups represented during training and did not test transfer to an unseen moisture level.

## 2. Materials and Methods

### 2.1. Experimental Materials and Preparation

The wheat cultivar used in this study was Zhengmai 119, collected from Yanjin County, Xinxiang City, Henan Province, China. All wheat originated from a single source batch. To eliminate potential insect eggs or larvae already present in the samples, the wheat was heat-treated in an oven at 60 °C for 2 h before the experiment, following heat-treatment methods used for stored-grain insects [16,17]. Wheat moisture conditions were established using the saturated-salt static equilibrium method [18]. Saturated sodium bromide, sodium chloride, and potassium chloride solutions were used to establish three relative-humidity environments in sealed containers maintained at 28 °C. Relative humidity inside each container was monitored using a temperature–humidity sensor. The conditioned samples were designated as 13%, 16%, and 19% moisture groups, respectively. Each moisture condition was established in a separate conditioning container, with one container per condition and no independently replicated conditioning batches. Wheat samples were conditioned for two days. At the end of this period, repeated measurements using the moisture meter gave unchanged readings. Relative humidity in the containers was monitored and maintained at a stable level during conditioning and incubation. Moisture readings were obtained after conditioning and after completion of all hyperspectral acquisitions using an LDS-1G grain moisture meter (Lookout Photoelectric, Tianjin, China) operated with its default wheat setting. However, numerical readings were not retained, and no intermediate kernel-moisture measurements were performed. Accordingly, 13%, 16%, and 19% denote nominal moisture groups; the achieved moisture contents and their variability cannot be reported quantitatively, and moisture stability throughout incubation was not directly verified.

Active adult maize weevils with comparable vigor were selected for infestation. Healthy control and infestation groups were prepared at each moisture level. For each infestation group, approximately 200 g of wheat was exposed to 800 adult maize weevils for 48 h to allow oviposition on the kernels. Infestation was performed once for each moisture group, without independently repeated infestation batches. All adults were then removed, leaving wheat samples potentially containing maize weevil eggs. Healthy controls were never exposed to maize weevils but otherwise underwent the same heat pretreatment, moisture conditioning, incubation, and imaging procedures as the exposed groups. To obtain samples representing different developmental stages, the infested wheat was further incubated under the same controlled temperature and humidity conditions. Sampling days were operationally selected by considering both the sampling schedule reported for *S. oryzae* in reference [5] and the observed timing of substantial *S. zeamais* adult emergence in the present experiment. These considerations informed sampling but did not validate developmental stages. The four sampling times were labelled nominal egg, early larval, late larval, and pupal for descriptive purposes only. Subsequent adult emergence confirmed successful infestation but did not establish the developmental stage present at each earlier acquisition. The operational sampling days and nominal stage labels for each nominal moisture group are provided in Table S1.

### 2.2. Hyperspectral and Image Acquisition

For hyperspectral image acquisition, individual wheat kernels were arranged in a single layer in a flat sample container without touching or overlapping, ensuring accurate extraction of single-kernel regions of interest (ROIs). Kernels remained at fixed positions on their original trays and were scanned together with the trays at each acquisition, without being removed or rearranged. Multiple trays were used because of scanning capacity limitations; they represented acquisition groups rather than independent conditioning or infestation batches. At each nominal moisture level (13%, 16%, and 19%), healthy kernels were distributed across two trays and exposed kernels across eight trays, giving a total of 30 scanning trays across the three moisture conditions. At each acquisition time point, healthy-control and exposed-group trays were imaged separately on the same day. All trays had the same material and specifications, and acquisition settings were kept unchanged between tray scans. There was no fixed order of scanning the two groups, and no predefined randomization procedure was used. Near-infrared hyperspectral images were acquired using a GAIA SORTER hyperspectral imaging system (Dualix Spectral Imaging, Wuxi, China) over 853.70–1701.80 nm at a scanning speed of 0.5 cm s^−1^. Three-channel pseudocolor images were generated from the calibrated hyperspectral data using a subsequent Python processing pipeline, as described in Section 2.3. Both model inputs originated from the same hyperspectral acquisition; no independent visible-color images were acquired. In subsequent modeling, the mean spectrum of each single-kernel ROI was used as input to the spectral branch, while the corresponding pseudocolor image was used as input to the image branch for spectral image classification.

During sample incubation, hyperspectral images were acquired on days 1 and 2 and every two days thereafter (days 4, 6, 8, etc.). Records corresponding to the four sampling time points specified for each moisture condition in Table S1 were retained for model construction. After imaging, all scanned kernels from the infestation groups were continuously incubated and individually tracked. Monitoring continued until day 32 after adult removal, when few additional adults were emerging. A kernel was retained as an infested sample only when subsequent adult maize weevil emergence confirmed internal infestation; the spectra and corresponding pseudocolor images previously acquired from that kernel were then assigned to the infested class. Only kernels with recorded adult emergence were eligible for the final infested class. Kernels without recorded emergence were not reassigned to the healthy class. Because emergence counting was discontinued before exhaustive enumeration, complete counts of kernels with and without emergence were unavailable. The final dataset contained healthy and confirmed-infested samples at different developmental stages under the 13%, 16%, and 19% moisture conditions. Each nominal moisture group initially contained 784 tracked insect-exposed kernels. Before counting was discontinued, adult emergence had been recorded from 346, 290, and 343 kernels in the nominal 13%, 16%, and 19% groups, respectively. Counting was stopped after emergence-confirmed kernels substantially exceeded the 196 healthy controls. To obtain approximately balanced class sizes, 200 emergence-confirmed kernels per nominal moisture group were selected sequentially according to their positions on the trays, rather than randomly or by emergence time. Visible feeding marks were considered only when selecting the initial 784 exposed kernels per nominal moisture group for tracking, with preference given to kernels without obvious marks; they were not used when selecting the final 200 emergence-confirmed kernels. At least 146, 90, and 143 recorded emergence-confirmed kernels, respectively, were not included in the final dataset. For each moisture condition, the same 196 healthy kernels and 200 confirmed-infested kernels were sampled at four selected time points. This yielded 1584 paired spectral image records from 396 physical kernels per moisture condition, totaling 4752 records from 1188 physical kernels across the three moisture conditions. Each sample record contained hyperspectral data, the corresponding pseudocolor image, a moisture label, a developmental-stage label, and a healthy/infested class label for a single wheat kernel. The three single-moisture datasets were evaluated separately, while data from all three moisture groups were combined to form a mixed-moisture dataset for assessing classification under more complex moisture conditions.

### 2.3. Image Calibration and Mean Spectrum Extraction

White and dark reference images were acquired once at the beginning of each sampling day and used by the acquisition software to correct the sample images collected that day. References were not reacquired between tray scans. Tray changes were brief, and the system was neither restarted nor adjusted between scans; acquisition parameters remained unchanged. To reduce the effects of nonuniform illumination and camera dark current, the raw hyperspectral images were first calibrated using white and dark reference images as follows:

(1) $$R=\frac{I_{Raw}-I_{Dark}}{I_{White}-I_{Dark}}$$

In Equation (1), R denotes the calibrated reflectance image, $I_{Raw}$ denotes the raw hyperspectral image of the sample, $I_{Dark}$ denotes the dark reference image, and $\;I_{White}$ denotes the white reference image. The acquisition software applied the white- and dark-reference correction before ROI extraction and pseudocolor image generation.

After calibration, the region of interest (ROI) of each individual wheat kernel was extracted in ENVI 5.6. The kernel ROIs delineated in ENVI were used to construct a binary mask. The mask was applied to all spectral bands in ENVI to suppress the background before the hyperspectral cube was exported for Python processing. Individual kernels were identified as connected components of the binary mask. After screening, 256 consecutive valid bands were kept for each kernel. To obtain an input feature representing the overall spectral response of a single kernel, reflectance values for all valid pixels within the ROI mask were spatially averaged at each wavelength, yielding a 256-dimensional mean spectrum. This mean spectrum was used as the 1D input to the hyperspectral branch, while the corresponding image was used as the input to the image branch for subsequent deep learning model construction.

Three-channel pseudocolor images were constructed using bands centered at 1454.50, 1202.60, and 969.80 nm, assigned to the red, green, and blue channels, respectively. For each kernel, the bounding box of its connected component was expanded by six pixels on each side, subject to the image boundaries, and the corresponding three-band region was cropped from the masked hyperspectral data. Min–max scaling was performed separately for each crop, using a common minimum and maximum across all its pixels and channels to map values to 0–255. Each crop was then converted to an 8-bit unsigned integer image and resized to 64 × 64 pixels using OpenCV area interpolation. Pixel values were divided by 255 when loaded for modeling. Scaling was therefore performed per image, rather than per acquisition or using parameters fitted on the training set.

### 2.4. Asymmetric Dual-Stream Masked Autoencoder Fusion Network

**Figure 1:**
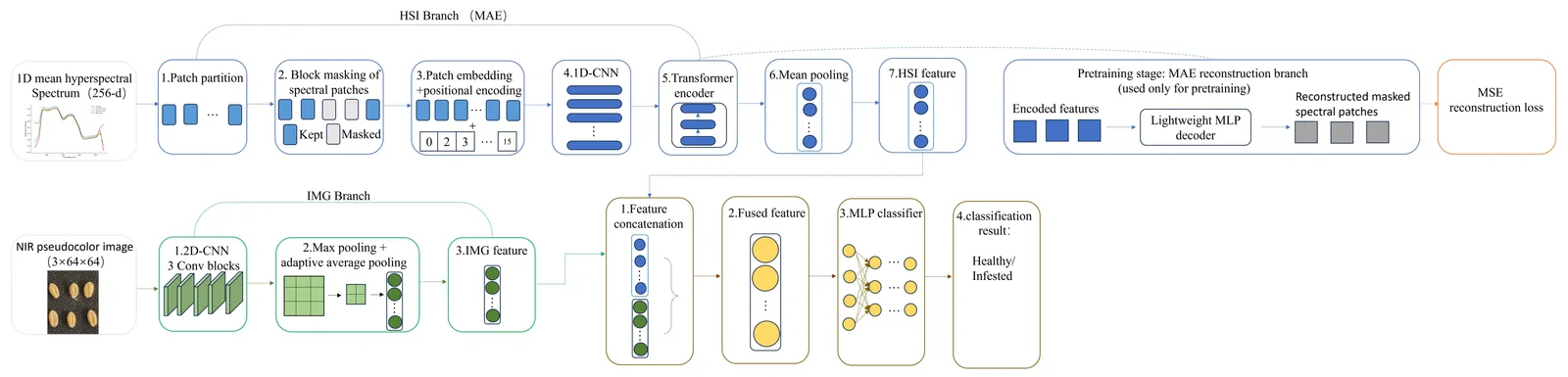
Architecture of ADS-MAFNet.

An Asymmetric Dual-Stream Masked Autoencoder Fusion Network (ADS-MAFNet) was developed to classify healthy and maize-weevil-infested wheat under varying moisture conditions. The model comprises an HSI branch, an IMG branch, and a feature-fusion classifier arranged in a parallel dual-stream architecture. The HSI branch takes the 256-dimensional 1D mean spectrum of a single kernel as input, uses a hybrid CNN–Transformer to extract spectral features, and employs MAE pretraining to learn relationships among spectral bands. The IMG branch takes the corresponding single-kernel pseudocolor image as input and uses a 2D-CNN to learn image representations from the selected-band spatial data. Each branch outputs a 128-dimensional feature vector; the two feature vectors are concatenated and passed to an MLP classifier for binary classification. The overall architecture is shown in Figure 1.

The 256-dimensional spectrum was divided into 16 contiguous patches, each containing 16 bands. During MAE pretraining, the masking-ratio parameter was set to 0.45, resulting in ⌊16 × 0.45⌋ = 7 masked patches. Thus, 112 of the 256 bands were masked, corresponding to an effective masking proportion of 43.75%. The seven masked patches formed one contiguous block, with its starting position randomly selected for each sample. Their embeddings were replaced with a learnable mask token. All 16 tokens, including the mask tokens, were processed by the encoder.

The encoder comprises a linear patch embedding from 16 to 128 dimensions, fixed sinusoidal positional encoding, a local 1D-CNN, and a Transformer encoder. The CNN operates along the patch-token sequence, whereas the Transformer models relationships among spectral patches. The local CNN comprised two one-dimensional convolutional layers operating along the patch-token sequence, with a residual addition to the embedded tokens. Each spectral convolution used 128 channels, a kernel size of 3, and padding of 1, followed by batch normalization and GELU activation. The two Transformer encoder layers used an embedding dimension of 128, four attention heads, and a feedforward dimension of 512. The Transformer used pre-normalization and GELU activation. Encoder dropout was 0.1 during MAE pretraining and 0.2 during supervised training. Layer normalization was applied to the output tokens before mean pooling. A lightweight MLP decoder reconstructs the masked original spectral patches. The token-wise decoder comprised linear layers of sizes 128–128 and 128–16, with GELU activation and dropout of 0.1 between the layers. Mean squared error (MSE) is used as the reconstruction loss and is calculated only over the masked patches:

(2) $$L_{rec}\;=\frac{1}{BMP}\sum_{b=1}^{B}\sum_{m\in \Omega_{b}}\sum_{p=1}^{P}{\;(x_{bmp}-{\hat{x}}_{bmp})}^{2}$$

In Equation (2), B is the batch size, $\Omega_{b}$ is the set of masked patches for sample b, M = 7 is the number of masked patches per sample, and P = 16 is the number of bands in each patch. The terms $x_{bmp}$ and ${\hat{x}}_{bmp}$ denote the original and reconstructed values, respectively. After pretraining, the decoder is discarded. During supervised fine-tuning and testing, the complete unmasked spectrum is processed by the pretrained CNN–Transformer encoder without masking. Mean pooling is then applied to obtain the spectral feature vector for subsequent fusion with the IMG branch features.

The IMG branch uses a lightweight 2D-CNN with an input size of 3 × 64 × 64. It contains three convolutional blocks, each consisting of a convolutional layer, batch normalization, and a GELU activation function. The number of channels increases sequentially from 3 to 32, 64, and 128. All convolutions used 3 × 3 kernels with unit stride and padding. The first two blocks were followed by 2 × 2 max pooling. The third block was followed by adaptive average pooling to 1 × 1, flattening, dropout of 0.2, and a 128–128 linear projection with GELU activation, producing a 128-dimensional image feature vector.

During feature fusion, the feature vectors output by the hyperspectral and IMG branches are directly concatenated:

(3) $$z_{f}\;=[z_{hsi}\;;z_{img}\;]$$

In Equation (3), $z_{hsi}$ and $z_{img}$ denote the hyperspectral and IMG feature vectors, respectively; the semicolon denotes vector concatenation. The concatenated feature vector is 256-dimensional and is subsequently passed to an MLP classifier to predict the two classes: healthy and infested. The classifier comprised LayerNorm followed by linear layers of sizes 256–128, 128–64 and 64–2, with GELU activation and dropout of 0.1 after each hidden layer.

**Figure 2:**
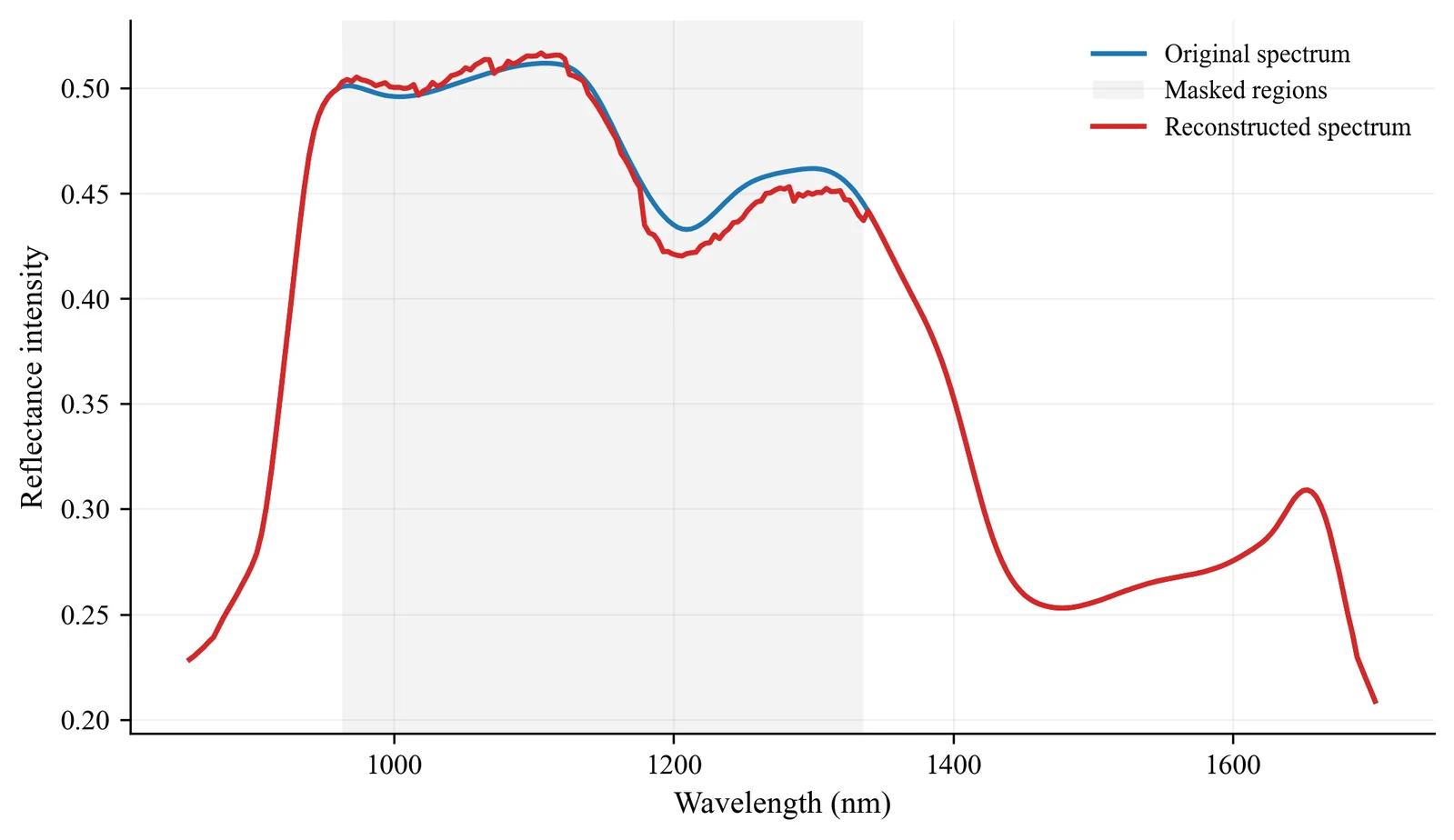
Visualization of MAE-based hyperspectral reconstruction.

To visualize the ability of MAE pretraining to reconstruct one-dimensional hyperspectral sequences, one infested kernel from the nominal 13% moisture group at the third selected sampling time point was used as an example. The input spectrum contained 256 bands. During pretraining, continuous block masking with a mask ratio of 0.45 was applied, meaning that approximately 45% of the spectral patches were randomly masked. The CNN–Transformer encoder processed the complete token sequence, with the embeddings of masked patches replaced by learnable mask tokens, and a lightweight MLP decoder reconstructed the masked spectral patches. Figure 2 shows the original spectrum, the masked spectrum, and the MAE-reconstructed spectrum, illustrating the model’s ability to learn both local continuity and global contextual relationships in the spectral sequence.

The original, masked, and completed spectra are shown. In the completed spectrum, unmasked values are copied from the input, whereas masked values are supplied by the decoder. Shading indicates the masked interval, comprising seven contiguous patches (112 bands; approximately 963.0–1335.8 nm), rather than spectral variability or a confidence interval.

### 2.5. Comparative Models and Ablation Experiments

To evaluate the effectiveness of the proposed model, comparative and ablation experiments were conducted. The comparison included 1D-CNN, Transformer, CNN–Transformer, HSI-MAE, 2D-CNN, PLS-DA and the proposed ADS-MAFNet fusion model. The 1D-CNN, Transformer, CNN–Transformer, and HSI-MAE models used 1D mean spectra as input; the 2D-CNN used the pseudocolor images alone; and ADS-MAFNet used both HSI and IMG representations. All models were evaluated using the same data-partitioning strategy and performance metrics. The CNN–Transformer baseline processed the same mean spectrum through parallel CNN and Transformer pathways and combined their features using learned gating weights. This differed from the convolution-followed-by-Transformer encoder used in ADS-MAFNet. Detailed architectural specifications of ADS-MAFNet and all comparison models, including layer configurations, feature dimensions, activation functions, pooling operations, dropout settings, and classifier structures, are provided in Table S7. Training, MAE pretraining, and validation-based model-selection settings are summarized in Table S8.

PLS-DA was implemented using PLS regression with class labels of 0 for healthy kernels and 1 for infested kernels. Centering and scaling were fitted on the training data. The number of latent variables was selected from 1, 2, 3, 5, 8, 10, 15, and 20 by maximizing validation F1 at a threshold of 0.5; ties favored fewer latent variables. Continuous prediction scores were used for ROC analysis and subsequent validation-based threshold selection.

To examine the contribution of each model component, four ablation variants were evaluated: the model without MAE, an HSI single-branch model, an IMG single-branch model, and the complete ADS-MAFNet. The model without MAE assessed the contribution of self-supervised pretraining to the fusion model; the HSI single-branch model assessed the independent classification ability of the hyperspectral branch; the IMG single-branch model assessed the contribution of the image branch; and the complete model assessed the effectiveness of spectral image fusion.

The HSI-MAE comparator in Table 1 and the HSI-only ablation in Table 2 shared the CNN–Transformer spectral-encoder architecture and a 128–128–64–2 MLP head with dropout of 0.2. Their training protocols differed: the standalone comparator used a learning rate of 3 × 10^−4^ without initial encoder freezing, whereas the HSI-only ablation used 5 × 10^−5^ and froze encoder parameters for the first 10 epochs. Both used training-only MAE pretraining, but their learned pretrained weights were not assumed identical. Likewise, the 2D-CNN comparator and IMG-only ablation shared the same image encoder and MLP-head architecture, but used learning rates of 3 × 10^−4^ and 5 × 10^−5^, respectively. The corresponding results therefore reflect different training settings, not exclusively changes in modality or network structure.

### 2.6. Training Parameters and Evaluation Metrics

All deep learning models were implemented in PyTorch 2.8.0+cu129 [19]. Model training was performed using Python 3.13.7, PyTorch 2.8.0+cu129, NumPy 2.1.2, pandas 2.3.2, SciPy 1.16.2, and scikit-learn 1.8.0. Local kernel identifiers were reused across acquisition sources and moisture conditions. A physical kernel was therefore identified in the retained metadata by the combination of moisture condition, class label, acquisition source (Source_Folder), and local kernel identifier (Grain_ID). The four sampling-time records associated with this combination represented repeated observations of the same kernel. For each single-moisture experiment, records were first restricted to the corresponding moisture condition; for the mixed-moisture experiment, records from all three moisture conditions were combined. The implemented allocation key consisted of the class label and local kernel identifier. This key was coarser than physical-kernel identity: distinct kernels sharing the same key were assigned together to one subset. Class-stratified group splitting targeted approximately 65%, 15%, and 20% for training, validation, and testing, respectively. Auditing the saved manifests for all 40 partitions confirmed that all four observations of each physical kernel remained in the same subset, with no physical-kernel overlap among training, validation, and test subsets. All models used identical partitions within each condition and random seed. Shared local identifiers across moisture conditions do not establish biological pairing. The partition seeds were 40, 41, 42, 43, 44, 45, 46, 47, 48, and 49. MAE pretraining used only the training set from the current partition; neither the validation nor test set was used. The IMG branch likewise did not load weights pretrained on the full dataset. The test set was used only for final model evaluation. All spectral neural models reported in Table 1 used the original 256-dimensional mean spectra without SNV or bandwise standardization. PLS-DA used internal centering and scaling fitted exclusively on the corresponding training set.

For supervised classification, cross-entropy loss and the AdamW optimizer [20] were used, with a batch size of 32 and a maximum of 100 training epochs. For models incorporating MAE pretraining, MSE was used as the spectral reconstruction loss during pretraining. During classification fine-tuning, only the MAE encoder was retained and the decoder was discarded. During the first 10 epochs of main-model training, the hyperspectral MAE encoder was frozen; it was then unfrozen for supervised fine-tuning. The IMG branch was trained under supervision using the current training split.

Each model was evaluated across ten repeated data partitions and training runs, and results are reported as mean ± sample standard deviation (SD). These summaries describe variation across partitions and training runs and do not represent ten independent experimental replicates. Performance was assessed using accuracy, precision, recall, and F1-score. Accuracy represents the proportion of all samples classified correctly; precision represents the proportion of predicted infested samples that were truly infested; recall represents the proportion of truly infested samples correctly identified; and F1-score is the harmonic mean of precision and recall. Sensitivity was calculated as TP/(TP + FN), specificity as TN/(TN + FP), and balanced accuracy as the mean of sensitivity and specificity. Per-run classification rules, thresholds, confusion counts, class-specific record counts, and unique-kernel counts are provided in Table S4. Sensitivity and specificity use the numbers of infested and healthy test records as their respective denominators.

For the ablation experiments, all four variants used a learning rate of 5 × 10^−5^, a batch size of 32, a maximum of 100 epochs, and an early-stopping patience of 20 epochs. The complete and spectral-only models used MAE pretraining and froze the encoder parameters for the first 10 supervised epochs. The no-MAE model was trained from scratch without encoder freezing. Classifier dropout was 0.1 for the complete and no-MAE models and 0.2 for the single-branch models.

Supervised training used AdamW with a weight decay of 1 × 10^−4^, a batch size of 32, and a maximum of 100 epochs. The learning rate was fixed at 5 × 10^−5^ for ADS-MAFNet and its ablation variants and at 3 × 10^−4^ for the five standalone deep learning comparators. No learning-rate scheduler was used. Training stopped after 20 consecutive epochs without a strict improvement in validation F1. The checkpoint with the highest validation F1 was retained; ties retained the earlier checkpoint. MAE pretraining used only the current training subset for 100 epochs with a batch size of 128, a learning rate of 1 × 10^−3^, weight decay of 1 × 10^−4^, and dropout of 0.1; the final pretraining encoder was transferred to supervised training, where encoder dropout was 0.2. For ADS-MAFNet and the HSI-only ablation, encoder parameters were frozen for the first 10 supervised epochs, and AdamW was reinitialized when all encoder parameters were unfrozen at epoch 11. No encoder freezing was used for the standalone HSI-MAE comparator. The neural models were evaluated using fixed, model-specific hyperparameter settings rather than a common validation-search budget. Identical data partitions and evaluation metrics controlled differences in evaluation data, but did not establish equivalent tuning effort. The comparisons therefore characterize performance under the reported training settings rather than the relative performance of fully optimized model architectures.

Paired model differences were assessed retrospectively using the existing group-disjoint test partition generated with random seed 40. The fitted models, test-record lists, and original classification rules were held fixed: argmax for neural networks and a score threshold of 0.5 for PLS-DA. A paired cluster bootstrap with 10,000 resamples was performed within the observed moisture, class, and tray strata. All four sampling-time observations from each sampled kernel were retained, and identical resampling indices were used for compared models. The 95% percentile intervals were conditional on the fitted models and fixed classification rules and did not account for retraining or between-container variability. Paired differences in F1-score and balanced accuracy, with their conditional 95% intervals, are reported in Table S3.

As a supplementary operating-point analysis, a classification threshold was selected for each fitted model by maximizing F1-score on its corresponding validation set. Ties were resolved by higher specificity, followed by proximity to 0.5 and then the larger threshold. The selected threshold was fixed before test evaluation and applied unchanged across moisture and sampling-time subgroups. This analysis used the same fitted models as the primary analysis. Test sensitivity, specificity, balanced accuracy, and F1-score at the validation-selected thresholds are summarized as means ± sample standard deviations across ten runs in Table S2. Conditional 95% intervals for sensitivity and specificity at the validation-selected thresholds on the seed-40 test partition are reported in Table S2b. These intervals were obtained using 10,000 kernel-cluster bootstrap resamples within moisture, class, and tray strata, retaining all four observations per sampled kernel while holding the fitted models and thresholds fixed. They exclude retraining and between-tray or between-batch uncertainty.

Image augmentation was applied online during training only. Horizontal and vertical flips were applied independently with probability 0.5. With probability 0.35, image intensities were transformed as I’ = aI + b, where a and b were sampled independently from normal distributions with means of 1 and 0 and standard deviations of 0.08 and 0.025, respectively. With probability 0.25, a rectangular region with width and height each ranging from 8% to 18% of the image dimensions was filled with the corresponding whole-image channel means. Values were clipped to [0, 1]. No stochastic augmentation was applied during validation or testing.

A supplementary analysis examined the sensitivity of Transformer performance to input scaling and optimization settings. The model used the same CLS-based architecture and dropout rate of 0.2 as the Transformer in the main comparison. Bandwise standardization parameters were estimated exclusively from the training subset of each partition and then applied to its validation and test subsets. Two learning rates, 5 × 10^−5^ and 3 × 10^−4^, were evaluated using the corresponding validation subset. Training used batches of up to 64 samples, a maximum of 60 epochs, and an early-stopping patience of 12, subject to a minimum of 20 epochs. Model selection prioritized validation F1-score at a fixed classification threshold of 0.5, with lower validation cross-entropy used to resolve ties. Test data were not used for configuration selection. This analysis was reported separately from the main model comparison. Training configurations and validation-based selection procedures are provided in Table S5, and test performance of the original and supplementary Transformer configurations is reported in Table S6.

## 3. Results

### 3.1. Spectral Analysis Under Different Moisture Conditions

**Figure 3:**
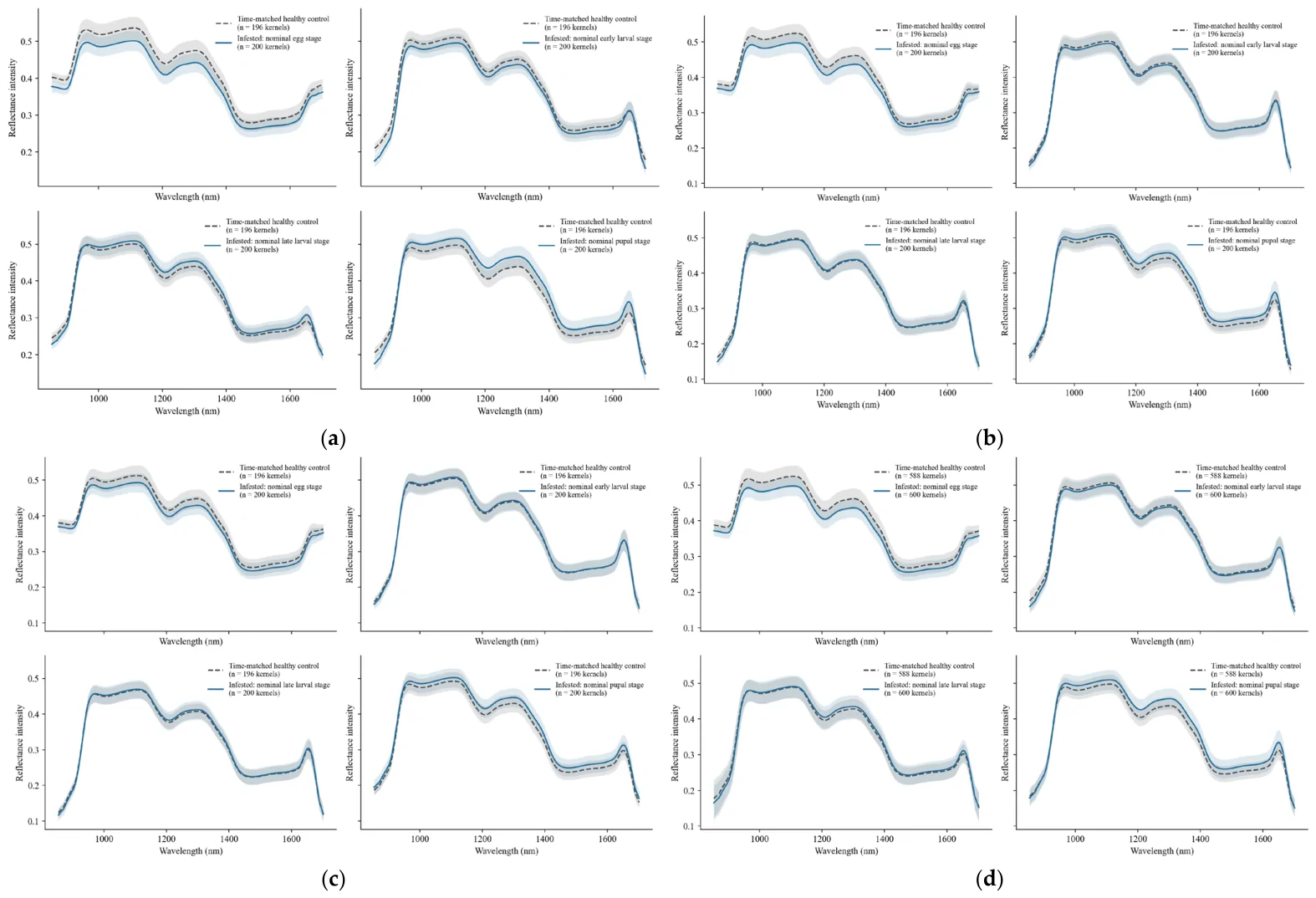
Mean spectra of infested kernels and time-matched healthy controls under (**a**) 13%, (**b**) 16%, (**c**) 19%, and (**d**) mixed-moisture conditions.

To examine changes in spectral response during hidden maize weevil infestation, mean spectral curves were plotted for samples at four operationally defined sampling times under 13%, 16%, 19%, and mixed-moisture conditions (Figure 3). The four stages correspond to the nominal egg, early larval, late larval, and pupal stages, respectively.

Within each moisture group, the panels show the nominal egg, early larval, late larval, and pupal sampling times from top left to bottom right. Blue solid and grey dashed curves represent infested kernels and healthy controls, respectively. Shading indicates ±1 sample standard deviation across kernels at each wavelength. Each sampling time includes 200 infested and 196 healthy kernels per single-moisture condition, or 600 infested and 588 healthy kernels after pooling the three moisture conditions. The same kernels were followed across sampling times.

Figure 3 shows differences in mean reflectance between infested kernels and time-matched healthy controls, together with substantial overlap in their spectral variability. The magnitude and direction of these differences vary with wavelength, moisture condition, and sampling time. Changes near the spectral limits are also present in the healthy controls, so they cannot be attributed specifically to infestation from these plots alone. Their origin remains unresolved. These descriptive spectral patterns do not by themselves establish classification separability.

Under mixed-moisture conditions, the curves were influenced simultaneously by infestation stage and moisture-background variation, resulting in more complex spectral relationships.

These results indicate that wheat samples at four operationally defined sampling times show some differences in their mean spectra, but these differences are not stable and are readily affected by moisture conditions. Classification analyses were therefore conducted to compare mean spectra, IMG features, and their combination.

### 3.2. Comparison of Classification Models

To compare classification models for hidden maize weevil detection under varying moisture conditions, 1D-CNN, Transformer, CNN–Transformer, HSI-MAE, 2D-CNN, PLS-DA, and the proposed ADS-MAFNet were evaluated under the three single-moisture conditions (13%, 16%, and 19%) and the mixed-moisture condition. Each model was evaluated across ten kernel-grouped train–validation–test partitions, with identical partitions used for all models. Performance is reported as mean ± sample standard deviation. Because kernels overlapped across partitions, these summaries describe partitioning and training variability rather than variation among independent biological replicates.

Classification performance under the four moisture conditions is summarized in Table 1.

As shown in Table 1, classification performance varied markedly with moisture condition. At 13% moisture, the models generally performed well, and both the hyperspectral models and the fusion model achieved high classification performance. At 16% and 19% moisture, however, the hyperspectral-only models deteriorated substantially, with the largest decline observed at 19% moisture. This pattern was observed for most spectral deep learning models, whereas PLS-DA retained comparatively high performance.

Differences among representations were also evident. Under low-moisture conditions, the performance gap between hyperspectral-only models and the fusion model was small, and the CNN–Transformer result at 13% moisture was already close to that of ADS-MAFNet. As moisture conditions became more challenging, the advantage of hyperspectral-only models diminished, whereas the relative performance of 2D-CNN and ADS-MAFNet remained more stable. ADS-MAFNet achieved higher mean accuracy and F1-score than the evaluated deep learning comparators under the 19% and mixed-moisture conditions. However, PLS-DA achieved higher mean accuracy and F1-score than ADS-MAFNet under all four conditions, showing that spectral image fusion did not outperform the conventional spectral baseline in this comparison.

Paired differences in F1-score and balanced accuracy, together with their conditional 95% intervals, are presented in Table S3. The supplementary Transformer analysis showed substantial sensitivity to the evaluated training settings. Mean test AUC increased from 0.557 to 0.928 at 13%, from 0.506 to 0.783 at 16%, from 0.498 to 0.602 at 19%, and from 0.537 to 0.814 under mixed moisture. All-positive predictions were eliminated in the supplementary runs at 13%, 16%, and mixed moisture, but persisted in two of ten runs at 19%. Full test metrics are reported in Table S6. Because input scaling and other training settings differed, these changes cannot be attributed to standardization alone.

**Figure 4:**
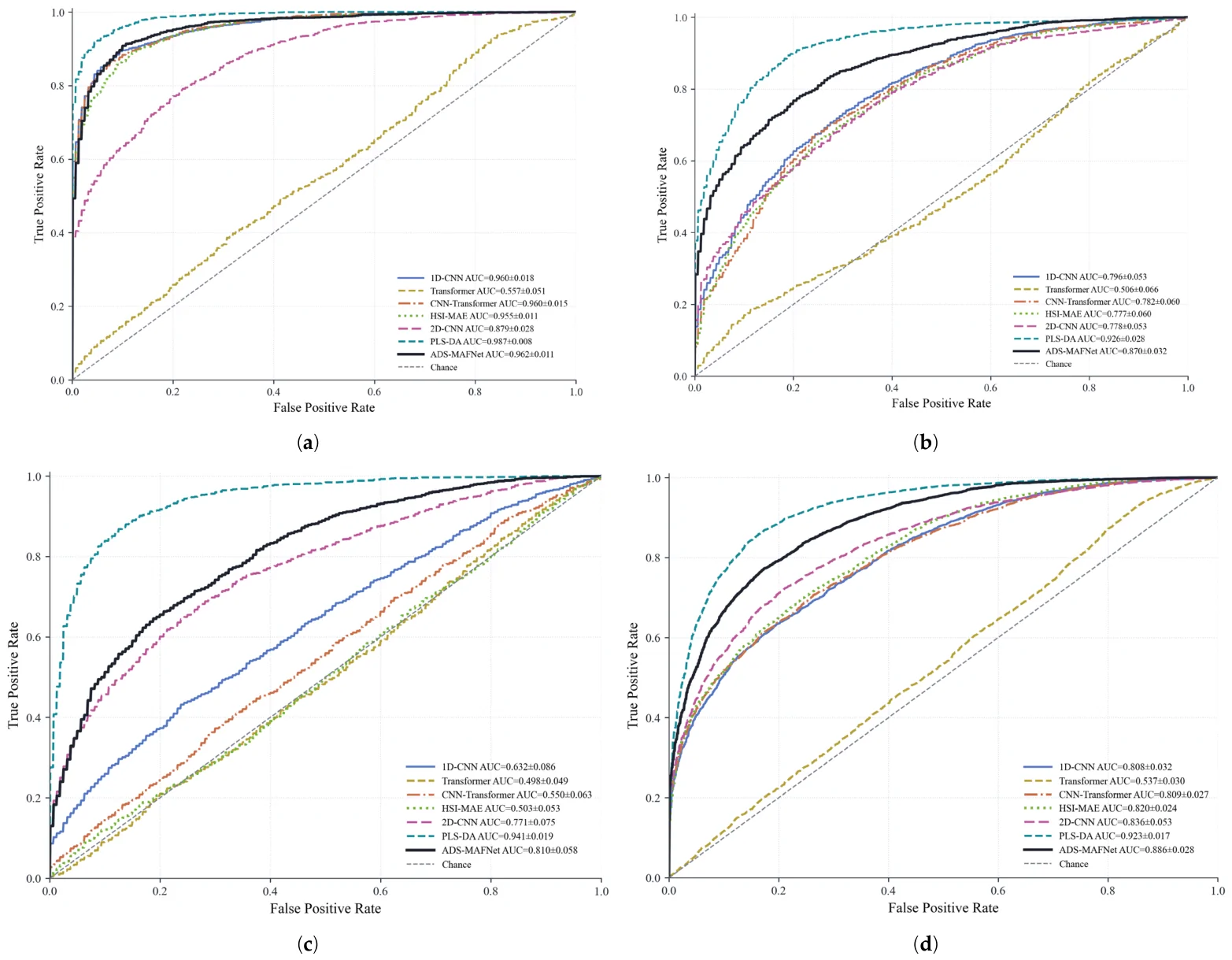
Mean ROC curves of different models under different moisture conditions: (**a**) 13% moisture; (**b**) 16% moisture; (**c**) 19% moisture; (**d**) mixed moisture.

To further evaluate the ability of the models to discriminate healthy and infested samples under different moisture conditions, receiver operating characteristic (ROC) curves were plotted for the 13%, 16%, 19%, and mixed-moisture datasets, as shown in Figure 4.

For each model in Figure 4, an ROC curve was calculated for each of the 10 random-seed runs. The true-positive rate (TPR) was interpolated onto a common false-positive-rate (FPR) grid and then averaged, while the AUC shown in the legend is the mean ± SD across the 10 runs. Overall, the ROC results were consistent with the classification metrics in Table 1. At 13% nominal moisture, the ROC curves of the spectral models other than the Transformer clustered near the upper-left corner, indicating strong discrimination within this condition. Most spectral neural comparators showed lower discrimination in the higher nominal moisture groups, whereas PLS-DA retained high discrimination across all three nominal moisture groups. In contrast, 2D-CNN and ADS-MAFNet retained more favorable ROC profiles under high-moisture and mixed-moisture conditions, with ADS-MAFNet achieving the highest mean AUC among the evaluated neural models, while PLS-DA achieved the highest mean AUC overall. These results further indicate that IMG features can complement mean hyperspectral information when the moisture background is complex, thereby improving overall discrimination.

### 3.3. Ablation Experiment Results

To assess the effects of MAE pretraining and the HSI and IMG branches on model performance, ablation experiments were conducted on the mixed-moisture dataset. Training and testing were repeated with 10 random seeds, and the results are presented in Table 2.

ADS-MAFNet achieved the highest ten-run mean accuracy and F1-score among the ablation variants. Compared with direct supervised training without MAE, the complete model increased accuracy and F1-score by 1.52 and 1.01 percentage points, respectively. MAE pretraining was associated with modest numerical gains under the evaluated training settings; these descriptive differences do not establish a consistent pretraining benefit. In the fixed-partition analysis, the conditional 95% intervals for F1 differences between the complete model and the no-MAE and IMG-only variants included zero (Table S3).

The ten-run mean accuracy and F1-score of the complete model exceeded those of both single-branch variants, with larger numerical differences than those observed relative to the no-MAE model. Relative to the HSI single-branch model, the complete model improved Accuracy and F1-score by 8.77 and 6.17 percentage points, respectively; relative to the IMG single-branch model, the corresponding gains were 4.78 and 4.00 percentage points. These ten-run mean differences suggest a potential benefit from combining the representations, but do not establish a consistent improvement over IMG-only or identify the features responsible for the differences.

The IMG-only branch showed relatively high recall but lower precision, indicating a greater tendency to identify samples as infested and, consequently, to misclassify some healthy samples as infested. The complete fusion model increased precision while retaining relatively high recall, producing a more balanced classification outcome. Combining the branches was associated with larger ten-run mean differences than MAE pretraining, but a consistent advantage over IMG-only was not established.

### 3.4. Classification Across Nominal Moisture Groups and Sampling Times

To characterize performance across nominal moisture groups and operational sampling times, ADS-MAFNet results were summarized for each group–time combination, as shown in Table 3.

As shown in Table 3, classification performance varied across nominal moisture groups and operational sampling times. At the nominal egg and early larval sampling times, accuracy and F1-score were generally lower in the nominal 16% and 19% groups than in the nominal 13% group. These descriptive differences do not isolate the effects of moisture from other differences among the experimental groups. Performance at the nominal late larval sampling time was lower in the nominal 16% and 19% groups than in the nominal 13% group, but remained higher than at the earlier sampling times. The pupal stage was the most stable across moisture conditions and retained relatively high performance even at 19% moisture and under mixed-moisture conditions, showing comparatively high classification performance at the nominal pupal sampling time within the investigated datasets.

Within each nominal moisture group, performance differed among operational sampling times; infestation severity was not directly measured. At 13% moisture, all stages were classified relatively well, and the gap between early and later stages was limited. At 16% and 19% moisture, performance at the egg and early larval stages decreased more sharply, whereas the late larval and pupal stages remained at higher levels, with larger differences between earlier and later sampling times in the nominal 16% and 19% groups. A similar pattern occurred under mixed-moisture conditions: the pupal stage performed best, followed by the late larval stage, while the egg and early larval stages showed lower performance. Thus, the performance degradation under varying moisture conditions was not uniform across stages but was concentrated primarily in the egg and early larval stages, indicating that detection of weak signals from early hidden infestation remains the principal bottleneck of this task.

### 3.5. Confusion Matrix Analysis

The selected random seeds are 48, 40, 45, and 40, respectively, with corresponding test sample sizes of 229, 237, 227, and 237 kernels. For each sampling time, the run with the test F1-score closest to the ten-run mean was selected. Matrices show counts obtained using the original argmax classification rule.

**Figure 5:**
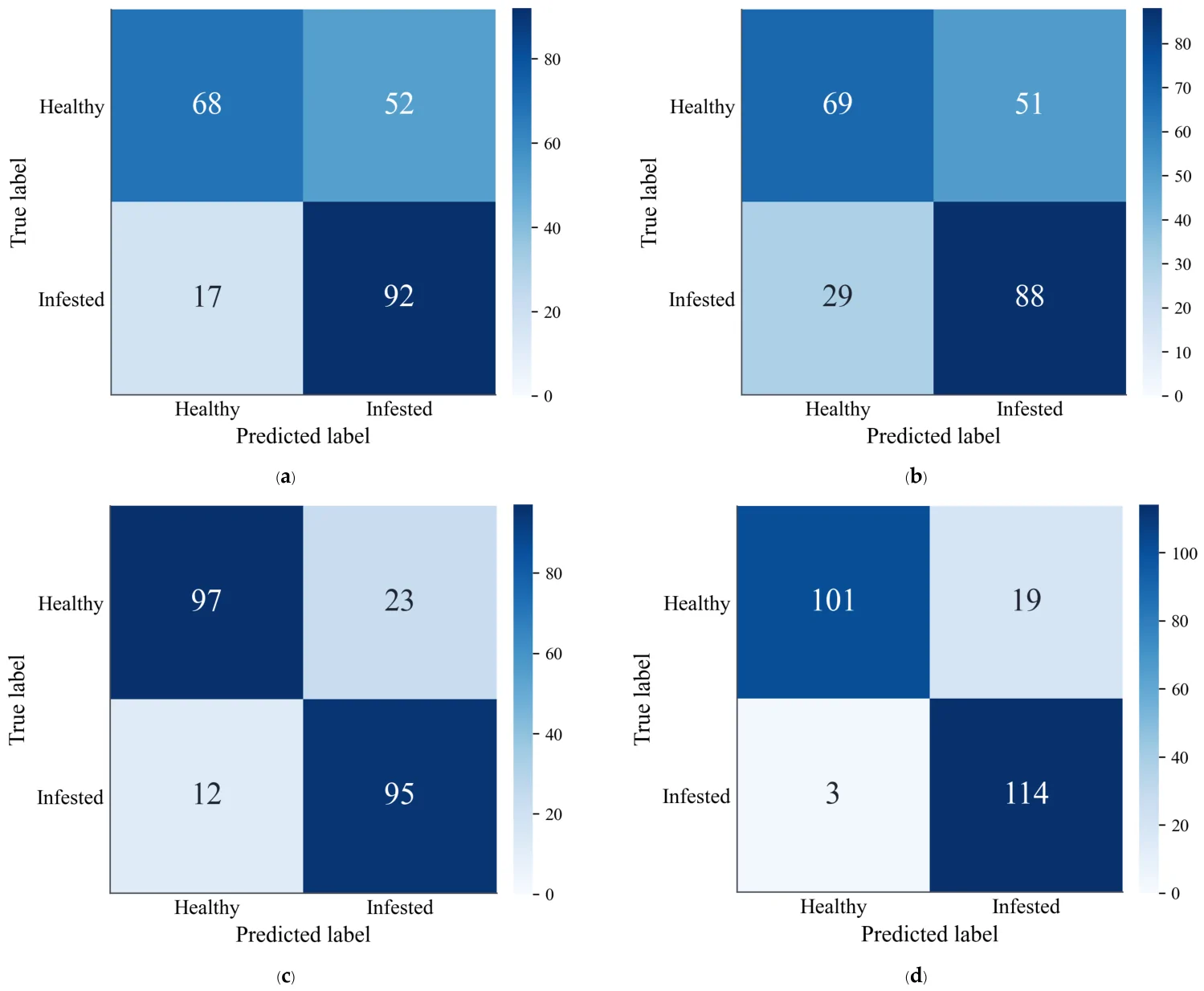
Representative confusion matrices of ADS-MAFNet under mixed-moisture conditions at the nominal (**a**) egg, (**b**) early larval, (**c**) late larval, and (**d**) pupal sampling times.

To examine the misclassification patterns of ADS-MAFNet under mixed-moisture conditions, Figure 5 presents representative confusion matrices at the nominal egg, early larval, late larval, and pupal sampling times. Representative runs were selected separately for each sampling time, resulting in different test partitions and sample sizes. Metrics calculated from these individual confusion matrices therefore need not equal the ten-run means in Table 3. In the representative egg-stage panel, 52 of 120 healthy kernels were classified as infested, corresponding to a specificity of 56.67%. This substantial false-positive burden pertains to the displayed run and is not the mean specificity across the ten runs.

Overall, the confusion matrices were consistent with the stage-specific metrics in Table 3: classification at the late larval and pupal stages was clearly better than at the egg and early larval stages. These results show that classification at the earlier operational sampling times was more difficult than at the later sampling times in the investigated mixed-moisture dataset.

## 4. Discussion

Spectral analysis under different moisture conditions showed some differences among infestation stages, but the overall separation was limited. Previous hyperspectral research on *S. oryzae* has suggested that infestation-related spectral differences may be associated with changes in kernel internal structure and composition [5]. Similar changes are a possible explanation for the performance differences among sampling times in the present study, but were not directly measured. Near-infrared hyperspectral responses are also highly sensitive to grain moisture; changes in moisture affect O–H-related absorption features, reflectance levels, and light-scattering behavior [21,22]. Moisture-related spectral variation may contribute to classification difficulty [23], but the present data do not isolate its effect from other differences among the experimental groups. Trays had the same material and specifications, acquisition settings were unchanged, and background masking was applied before image extraction. These controls reduced potential acquisition differences, but the two classes were still scanned separately. Class-associated acquisition effects were not independently evaluated and therefore cannot be fully excluded; their presence has not been demonstrated. The extent to which separate acquisition contributed to the observed classification performance remains unknown.

Previous studies have established an important basis for hyperspectral detection of hidden pests in wheat. Yan et al. [5] investigated hidden rice weevil infestation in wheat kernels using hyperspectral data acquired over 850–1700 nm at a moisture content of 13.0 ± 0.5%. Their best SVM models achieved classification accuracies above 94% at different infestation times, indicating that under a single, relatively stable moisture condition, mean spectra can contain sufficient information for discriminating hidden pest infestation. Singh et al. [11] combined short-wave near-infrared hyperspectral imaging with digital color imaging for identifying insect-damaged wheat kernels, demonstrating the complementary value of spectral information and image-based morphology and texture. In the present study, most spectral deep learning models performed less well in the nominal 16% and 19% groups than in the nominal 13% group, whereas PLS-DA retained comparatively high performance across groups. Spatial image information may complement mean spectral data [24,25], but the present experiments do not establish whether morphology, texture, wavelength-dependent intensity, or acquisition-related features account for the observed performance differences.

Differences among the models also reflect how the available features are used under varying moisture conditions. Under the reported training settings, the 1D-CNN and CNN–Transformer performed less well in the nominal 16% and 19% groups than in the nominal 13% group. This pattern cannot be separated from the effects of preprocessing and model configuration. The original Transformer configuration is retained in Table 1 to document the fixed-protocol comparison, rather than as evidence of the best attainable Transformer performance. The improved discrimination in the supplementary analysis shows that the original result underestimated performance achievable with alternative input processing and training settings. Consequently, the performance gap relative to this baseline cannot be interpreted as an inherent architectural advantage of ADS-MAFNet. The supplementary analysis does not resolve the absence of a matched hyperparameter-search budget across all models. In the supplementary analysis, the mixed-moisture mean AUC increased from 0.537 to 0.814 (Table S6). Because preprocessing and several training settings changed together (Table S5), this improvement cannot be attributed to standardization alone.

The stronger PLS-DA performance also indicates that the mean spectra retained useful predictive information under the evaluated conditions. PLS-DA and the spectral neural comparators used mean spectra extracted from the same kernel ROIs, although their scaling and training procedures differed. Their performance differences therefore do not, by themselves, demonstrate an error in spectral extraction. They instead show that the evaluated deep learning implementations did not exploit these inputs more effectively than PLS-DA. The limited number of physical kernels and the relationship between model complexity and the available training data are possible contributing factors, but their individual effects were not isolated. The supplementary Transformer results provide direct evidence of sensitivity to preprocessing and optimization settings, rather than proof of a specific feature-extraction failure.

Accordingly, the present results do not support replacing PLS-DA with ADS-MAFNet on the basis of predictive performance. The fusion model is evaluated as an exploratory representation-learning approach, and its higher ten-run means relative to selected neural baselines should not be interpreted as superiority over the conventional spectral comparator. By comparison, the 2D-CNN remained relatively stable at high moisture, although this does not establish that morphology or texture is less sensitive to moisture variation. However, because hidden maize weevil infestation develops mainly inside the kernel, image information alone cannot fully represent infestation status. The higher ten-run mean performance of the fusion model is compatible with complementary predictive information, but the fixed-partition comparison did not establish an advantage over IMG-only.

The ablation experiments provide a comparison of the predictive contributions of the two branches. Under mixed-moisture conditions, ADS-MAFNet achieved an accuracy of 79.40% and an F1-score of 79.44%, compared with 70.63% accuracy and 73.27% F1-score for the HSI-only ablation and 74.62% accuracy and 75.44% F1-score for the IMG single-branch model. These numerical differences are consistent with complementary predictive information from the two inputs under the evaluated settings, but do not establish the underlying mechanism. The smaller difference between the complete and no-MAE models does not establish a consistent benefit from MAE pretraining. Under the evaluated settings, combining the two branches was associated with larger numerical gains than introducing MAE pretraining.

Performance differences among nominal sampling times may be related to changes associated with infestation development. Lower performance at the egg and early larval stages may result from the relatively limited changes in internal tissue structure, nutrient composition, and external appearance at these stages, leaving insufficient discriminative information for the hyperspectral and image branches to learn [26]. Increasing internal damage is a possible explanation for the higher performance at later sampling times, but developmental stages and internal structural changes were not directly verified. The confusion matrices under mixed-moisture conditions show the corresponding performance pattern: misclassification was more frequent at the early stages, whereas classification was more stable at the later stages. The current model performed better at the later operational sampling times, while classification at the earlier sampling times remained more difficult.

This study was based primarily on single-kernel ROI mean spectra and experimentally infested wheat kernels generated under controlled conditions, with limited consideration of full spatial–spectral information and the complex factors present in real grain-storage environments [27,28]. No independent physicochemical measurements were performed to determine the causes of spectral differences among the nominal moisture groups. The present analysis therefore evaluates classification performance rather than establishing moisture-related physicochemical mechanisms. Each moisture condition was represented by one conditioning container. Matching random seeds does not establish biological pairing between distinct moisture groups. Consequently, moisture-related performance differences are interpreted descriptively rather than as independently replicated effects of moisture. Bootstrap stratification within observed trays does not estimate variability between trays or independent biological batches. The absence of independent conditioning and infestation replicates, together with separate imaging of healthy and exposed trays, means that acquisition-related effects associated with class cannot be ruled out. Kernel-level grouping prevents observations from the same kernel from crossing data subsets but does not remove effects shared by entire trays or batches. Retrospective labeling based on adult emergence restricted the positive class to kernels with successful infestation confirmed within the 32-day observation period. The results therefore do not establish performance for all insect-exposed kernels, including those with unsuccessful infestation, immature insect mortality, or emergence after the observation period. Future work should incorporate independently replicated batches with both healthy and infested samples, including samples from actual storage environments and multiple interacting environmental factors to further evaluate model generalization and practical robustness.

Selection based on adult emergence restricts the findings to infestations that supported adult development within the observation period. The initial consideration of visible feeding marks and subsequent nonrandom sequential selection may have introduced selection bias. Incomplete emergence counts prevent its extent from being quantified. Furthermore, the absence of independently replicated infestation treatments limits assessment of generalization to independent biological batches.

## 5. Conclusions

This study addressed the difficulty of detecting hidden maize weevil infestation in wheat under varying moisture conditions by constructing a paired near-infrared hyperspectral and pseudocolor image dataset covering three nominal moisture groups designated as 13%, 16%, and 19%, from which single-moisture and mixed-moisture datasets were derived, and by developing ADS-MAFNet, a spectral image fusion model incorporating MAE pretraining. Classification performance varied across the investigated nominal moisture conditions. The evaluated spectral deep learning models generally performed less well in the nominal 16% and 19% groups than in the nominal 13% group, whereas PLS-DA achieved higher mean accuracy and F1-score than ADS-MAFNet under all four conditions. Thus, an overall predictive advantage of the proposed deep fusion approach over the conventional spectral comparator was not demonstrated. Under the evaluated training settings, ADS-MAFNet achieved higher ten-run mean accuracy and F1-score than its HSI-only and IMG-only ablation variants on the mixed-moisture dataset, with accuracy, precision, recall, and F1-score values of 79.40%, 75.27%, 84.28%, and 79.44%, respectively. These results describe performance within the investigated laboratory conditions; the mixed-moisture evaluation does not establish transfer to an unseen moisture level. Classification was generally more difficult at the two earlier operational sampling times than at the two later sampling times. The approach warrants further evaluation using independently prepared batches and acquisitions in which both classes are represented within each batch and acquisition session. These findings apply to the experimental batches and acquisition conditions examined here; generalization to independently prepared batches remains to be established.

## Figures and Tables

**Table 1:** Classification performance of different models under different moisture conditions.

Moisture Condition	Model	Accuracy/%	Precision/%	Recall/%	F1-Score/%	Specificity/%	BA/%
13% moisture	1D-CNN	89.04 ± 2.92	90.73 ± 3.72	87.63 ± 5.91	89.00 ± 3.12	90.56 ± 4.30	89.10 ± 2.90
	Transformer	52.18 ± 3.30	51.67 ± 2.28	98.59 ± 2.98	67.73 ± 1.26	4.25 ± 9.00	51.42 ± 3.03
	CNN–Transformer	88.42 ± 3.43	92.35 ± 3.41	84.23 ± 4.79	88.05 ± 3.66	92.75 ± 3.39	88.49 ± 3.41
	HSI-MAE	88.16 ± 1.78	87.76 ± 3.70	89.50 ± 4.71	88.47 ± 1.77	86.81 ± 4.57	88.16 ± 1.79
	2D-CNN	77.49 ± 5.77	74.76 ± 7.05	86.44 ± 5.90	79.80 ± 3.69	68.19 ± 15.31	77.32 ± 5.87
	PLS-DA	93.43 ± 2.02	94.57 ± 2.41	92.41 ± 2.04	93.47 ± 1.96	94.50 ± 2.55	93.46 ± 2.03
	ADS-MAFNet	89.68 ± 1.22	89.23 ± 3.65	90.90 ± 3.26	89.95 ± 1.15	88.38 ± 4.14	89.64 ± 1.22
16% moisture	1D-CNN	70.30 ± 4.38	66.61 ± 5.31	82.70 ± 8.70	73.42 ± 3.96	57.88 ± 12.43	70.29 ± 4.36
	Transformer	49.79 ± 1.09	49.79 ± 1.09	100.00 ± 0.00	66.47 ± 0.98	0.00 ± 0.00	50.00 ± 0.00
	CNN–Transformer	70.04 ± 5.13	66.14 ± 5.42	83.48 ± 5.05	73.59 ± 3.47	56.69 ± 11.60	70.08 ± 5.06
	HSI-MAE	68.99 ± 5.66	65.55 ± 5.42	81.91 ± 7.55	72.49 ± 4.04	55.94 ± 14.68	68.92 ± 5.75
	2D-CNN	68.33 ± 4.93	64.83 ± 4.31	79.83 ± 12.48	71.09 ± 6.96	56.88 ± 9.32	68.35 ± 4.92
	PLS-DA	85.25 ± 3.50	84.39 ± 4.48	86.67 ± 3.47	85.45 ± 3.15	83.87 ± 5.42	85.27 ± 3.52
	ADS-MAFNet	77.84 ± 3.69	77.31 ± 6.33	79.62 ± 7.33	78.08 ± 3.85	76.25 ± 8.25	77.93 ± 3.61
19% moisture	1D-CNN	56.93 ± 5.86	53.99 ± 4.71	84.59 ± 10.36	65.38 ± 2.73	31.00 ± 20.18	57.79 ± 5.18
	Transformer	48.19 ± 2.81	48.19 ± 2.81	100.00 ± 0.00	64.99 ± 2.60	0.00 ± 0.00	50.00 ± 0.00
	CNN–Transformer	51.70 ± 5.04	50.09 ± 3.50	89.53 ± 13.16	63.84 ± 4.60	16.25 ± 16.77	52.89 ± 4.23
	HSI-MAE	48.90 ± 3.21	48.52 ± 3.04	93.74 ± 10.77	63.65 ± 3.63	7.19 ± 12.65	50.46 ± 1.14
	2D-CNN	66.90 ± 5.47	62.43 ± 5.83	79.91 ± 13.81	69.55 ± 6.99	54.00 ± 14.03	66.96 ± 5.47
	PLS-DA	87.04 ± 2.77	85.18 ± 5.02	88.95 ± 4.35	86.86 ± 2.75	85.38 ± 5.39	87.16 ± 2.56
	ADS-MAFNet	71.15 ± 3.88	66.71 ± 4.27	80.56 ± 14.23	72.28 ± 7.37	61.75 ± 11.64	71.16 ± 4.46
Mixed moisture	1D-CNN	70.36 ± 3.32	65.71 ± 4.91	79.57 ± 5.99	71.75 ± 3.63	62.25 ± 6.12	70.91 ± 2.92
	Transformer	47.56 ± 3.29	47.56 ± 3.29	100.00 ± 0.00	64.40 ± 3.09	0.00 ± 0.00	50.00 ± 0.00
	CNN–Transformer	70.48 ± 2.63	65.65 ± 4.29	80.16 ± 3.01	72.05 ± 2.35	61.71 ± 6.40	70.93 ± 2.38
	HSI-MAE	70.45 ± 2.13	65.15 ± 3.87	82.68 ± 7.63	72.56 ± 2.20	59.33 ± 8.98	71.01 ± 2.14
	2D-CNN	73.49 ± 6.05	70.01 ± 9.55	80.37 ± 6.64	74.26 ± 5.38	67.83 ± 12.18	74.10 ± 5.42
	PLS-DA	84.68 ± 2.23	81.97 ± 3.90	86.92 ± 2.34	84.32 ± 2.50	82.73 ± 3.23	84.82 ± 2.07
	ADS-MAFNet	79.40 ± 3.07	75.27 ± 4.26	84.28 ± 5.02	79.44 ± 3.77	74.77 ± 5.06	79.52 ± 3.09

Note: Values are percentages reported as means ± sample standard deviations across ten kernel-grouped partitions. Standard deviations describe variation across partitions and training runs. Recall is equivalent to sensitivity; BA denotes balanced accuracy. Deep learning models used argmax classification, whereas PLS-DA used a fixed score threshold of 0.5. The Transformer entry represents the original fixed training protocol, not a validation-optimized Transformer benchmark. Results from the supplementary configuration-selection analysis are reported separately in Table S6.

**Table 2:** Ablation results under mixed-moisture conditions.

Model Variant	Accuracy/%	Precision/%	Recall/%	F1-Score/%	Specificity/%	BA/%
ADS-MAFNet	79.40 ± 3.07	75.27 ± 4.26	84.28 ± 5.02	79.44 ± 3.77	74.77 ± 5.06	79.52 ± 3.09
Model without MAE	77.88 ± 2.06	73.02 ± 4.23	85.02 ± 4.29	78.43 ± 2.57	71.35 ± 5.58	78.19 ± 1.96
HSI single-branch model	70.63 ± 3.54	64.94 ± 4.54	84.62 ± 4.47	73.27 ± 2.20	57.83 ± 9.81	71.23 ± 3.12
IMG single-branch model	74.62 ± 3.26	69.81 ± 4.30	82.27 ± 4.45	75.44 ± 3.43	67.65 ± 5.55	74.96 ± 3.32

Note: Values are percentages reported as means ± sample standard deviations across ten kernel-grouped partitions. Standard deviations describe variation across partitions and training runs. Recall is equivalent to sensitivity; BA denotes balanced accuracy.

**Table 3:** Classification performance of ADS-MAFNet across nominal moisture groups and operational sampling times.

Moisture Condition	Nominal Stage	Accuracy/%	Precision/%	Recall/%	F1-Score/%	Specificity/%	BA/%
13% moisture	Egg stage	86.47 ± 3.75	87.96 ± 6.85	85.68 ± 3.19	86.63 ± 3.33	87.25 ± 7.77	86.47 ± 3.79
	Early larval stage	86.00 ± 1.42	85.65 ± 4.39	87.61 ± 6.09	86.36 ± 1.78	84.25 ± 5.66	85.93 ± 1.42
	Late larval stage	94.35 ± 2.75	97.76 ± 2.14	91.03 ± 5.61	94.17 ± 3.02	97.75 ± 2.19	94.39 ± 2.73
	Pupal stage	91.90 ± 3.52	87.01 ± 5.35	99.27 ± 1.62	92.65 ± 3.06	84.25 ± 6.98	91.76 ± 3.57
16% moisture	Egg stage	74.89 ± 4.02	76.10 ± 5.67	72.84 ± 5.71	74.25 ± 4.16	77.00 ± 6.95	74.92 ± 4.00
	Early larval stage	65.69 ± 6.70	66.00 ± 7.14	65.91 ± 14.63	65.05 ± 8.68	65.75 ± 11.79	65.83 ± 6.59
	Late larval stage	84.03 ± 3.28	82.67 ± 6.73	87.26 ± 9.06	84.35 ± 3.53	81.00 ± 8.99	84.13 ± 3.17
	Pupal stage	86.75 ± 5.05	83.80 ± 8.79	92.45 ± 6.06	87.51 ± 4.51	81.25 ± 11.32	86.85 ± 4.92
19% moisture	Egg stage	60.88 ± 3.73	57.35 ± 5.30	73.11 ± 15.64	63.58 ± 7.72	49.25 ± 12.59	61.18 ± 4.00
	Early larval stage	63.03 ± 5.23	59.20 ± 4.73	74.76 ± 17.37	65.22 ± 8.79	51.50 ± 14.59	63.13 ± 5.57
	Late larval stage	74.39 ± 5.57	70.27 ± 6.26	83.70 ± 17.06	75.11 ± 8.58	64.75 ± 16.14	74.23 ± 6.09
	Pupal stage	86.29 ± 5.58	84.03 ± 8.98	90.68 ± 14.03	85.98 ± 7.38	81.50 ± 12.43	86.09 ± 6.18
Mixed moisture	Egg stage	71.33 ± 3.87	66.51 ± 6.50	82.02 ± 5.45	73.10 ± 3.20	61.92 ± 9.76	71.97 ± 3.30
	Early larval stage	69.81 ± 5.42	66.54 ± 5.99	72.51 ± 10.66	69.20 ± 7.42	67.00 ± 6.63	69.75 ± 5.68
	Late larval stage	84.74 ± 2.94	81.34 ± 3.74	88.23 ± 7.58	84.44 ± 3.91	81.17 ± 6.23	84.70 ± 3.19
	Pupal stage	91.70 ± 1.95	88.96 ± 2.87	94.35 ± 4.44	91.49 ± 2.34	89.00 ± 3.92	91.67 ± 2.03

Note: Values are percentages reported as means ± sample standard deviations across ten kernel-grouped partitions. Standard deviations describe variation across partitions and training runs. Recall is equivalent to sensitivity; BA denotes balanced accuracy. Healthy controls were evaluated at the corresponding sampling times.

## Data Availability

The data presented in this study are available from the corresponding author upon reasonable request. The data are not publicly available at this stage because they form part of an ongoing graduate thesis and related unpublished research. The code used for preprocessing, data partitioning, model training, model selection, and evaluation has been provided to the editors and reviewers for peer-review assessment. It is not publicly available at present.

## Supplementary Materials

The following supporting information can be downloaded at https://www.mdpi.com/article/10.3390/foods15214000/s1. Table S1: Operational sampling days and nominal stage labels for each nominal moisture group; Table S2: Classification performance at validation-selected operating thresholds; Table S2b: Sensitivity and specificity on the fixed test partition with conditional 95% kernel-cluster bootstrap intervals; Table S3: Paired model differences with conditional 95% bootstrap intervals; Table S4: Per-run test counts, classification rules, thresholds, and performance metrics, provided as a separate CSV file; Table S5: Training configurations and validation-based selection procedures for the Transformer analyses; Table S6: Test performance of the original and supplementary Transformer configurations; Table S7: Architectural specifications of ADS-MAFNet and the comparison models; Table S8: Training, model selection, data partitioning, and classification settings.

## Abbreviations

The following abbreviations are used in this manuscript:

ADS-MAFNet	Asymmetric Dual-Stream Masked Autoencoder Fusion Network
HSI	Hyperspectral
HSI-MAE	Hyperspectral imaging masked autoencoder
IMG	Pseudocolor image
MAE	Masked autoencoder
MLP	Multilayer perceptron
MSE	Mean squared error
ROC	Receiver operating characteristic
ROI	Region of interest
SD	Standard deviation
CNN	Convolutional neural network
AUC	Area under the receiver operating characteristic curve
