# Supplementary material for: Detection of Hidden Maize Weevil Infestation in Wheat Under Varying Moisture Conditions Using an Asymmetric Dual-Stream Masked Autoencoder Fusion Network
Source: Foods. 2026 Nov 7;15(21):4000. doi: 10.3390/foods15214000 (PMC13763295; doi:10.3390/foods15214000)
Supplement: Supplementary file 1 [file foods-15-04000-s001.zip › Supplementary_Materials.pdf]

Supplementary Materials

Detection of Hidden Maize Weevil Infestation in Wheat under Varying Moisture Conditions Using an Asymmetric Dual-Stream Masked Autoencoder Fusion Network

Table S1. Operational sampling days after adult removal and nominal stage labels for each nominal moisture group

Nominal stage label	Nominal 13%	Nominal 16%	Nominal 19%
Nominal Egg stage	1	1	1
Nominal Early larval stage	14	12	10
Nominal Late larval stage	28	24	20
Nominal Pupal stage	32	30	26

Note: Values indicate days after adult removal. Sampling days were operationally selected by considering the sampling schedule for S. oryzae in reference [5] and the observed timing of substantial S. zeamais adult emergence in the present experiment. Stage labels are descriptive and were not directly validated at acquisition.

Table S2. Classification performance at validation-selected operating thresholds under different moisture conditions

Nominal Moisture condition	Model	Sensitivity	Specificity	Balanced accuracy	F1-score
13%	1D-CNN	87.36 ± 6.30	91.19 ± 2.64	89.28 ± 2.51	89.09 ± 2.96
13%	Transformer	95.22 ± 4.22	13.44 ± 6.78	54.33 ± 2.41	68.26 ± 1.57
13%	CNN-Transformer	85.91 ± 2.99	91.88 ± 3.63	88.89 ± 2.79	88.68 ± 2.80
13%	HSI-MAE	90.24 ± 4.23	85.62 ± 4.61	87.93 ± 1.80	88.38 ± 1.70
13%	2D-CNN	83.02 ± 6.49	73.88 ± 9.30	78.45 ± 3.74	79.73 ± 3.28
13%	PLS-DA	92.83 ± 2.86	94.62 ± 3.01	93.73 ± 2.15	93.76 ± 2.10
13%	ADS-MAFNet	91.15 ± 2.71	87.81 ± 4.06	89.48 ± 1.44	89.86 ± 1.28

Nominal Moisture condition	Model	Sensitivity	Specificity	Balanced accuracy	F1-score
16%	1D-CNN	83.66 ± 7.24	55.69 ± 10.28	69.68 ± 4.02	73.22 ± 3.67
16%	Transformer	94.79 ± 8.01	7.88 ± 8.31	51.33 ± 2.41	65.77 ± 2.66
16%	CNN-Transformer	84.89 ± 5.67	54.25 ± 10.67	69.57 ± 4.34	73.54 ± 3.07
16%	HSI-MAE	84.42 ± 9.73	52.56 ± 14.66	68.49 ± 5.80	72.67 ± 4.77
16%	2D-CNN	78.81 ± 10.46	58.88 ± 9.87	68.84 ± 4.82	71.37 ± 5.93
16%	PLS-DA	90.95 ± 3.17	79.19 ± 7.71	85.07 ± 3.94	85.93 ± 3.15
16%	ADS-MAFNet	80.17 ± 4.80	75.50 ± 7.06	77.84 ± 3.49	78.20 ± 3.38
19%	1D-CNN	85.77 ± 10.20	30.12 ± 21.05	57.95 ± 5.76	65.76 ± 2.64
19%	Transformer	99.13 ± 1.61	1.56 ± 2.11	50.34 ± 0.78	64.96 ± 2.60
19%	CNN-Transformer	93.09 ± 8.28	12.12 ± 13.07	52.61 ± 3.52	64.72 ± 3.17
19%	HSI-MAE	96.95 ± 4.57	4.25 ± 4.33	50.60 ± 1.47	64.62 ± 3.69
19%	2D-CNN	78.88 ± 14.56	55.25 ± 17.73	67.07 ± 5.87	69.46 ± 6.65
19%	PLS-DA	90.12 ± 4.93	83.38 ± 6.84	86.75 ± 2.56	86.67 ± 2.54
19%	ADS-MAFNet	81.65 ± 15.24	58.62 ± 13.58	70.14 ± 3.96	71.80 ± 7.08
Mixed	1D-CNN	85.83 ± 4.86	53.79 ± 8.18	69.81 ± 3.15	72.42 ± 3.31
Mixed	Transformer	97.25 ± 1.27	8.96 ± 3.99	53.11 ± 1.93	65.29 ± 3.51
Mixed	CNN-Transformer	86.29 ± 3.56	52.29 ± 7.30	69.29 ± 2.57	72.13 ± 2.68
Mixed	HSI-MAE	85.05 ± 7.23	55.67 ± 11.04	70.36 ± 3.13	72.77 ± 2.32
Mixed	2D-CNN	81.50 ± 5.93	66.73 ± 7.91	74.11 ± 5.10	74.56 ± 5.75
Mixed	PLS-DA	89.26 ± 2.62	79.73 ± 4.53	84.50 ± 2.25	84.27 ± 2.65

Nominal Moisture condition	Model	Sensitivity	Specificity	Balanced accuracy	F1-score
Mixed	ADS-MAFNet	85.28 ± 6.01	72.79 ± 5.80	79.03 ± 2.88	79.20 ± 3.56

Note: Values are percentages reported as means ± sample standard deviations across ten kernel-grouped partitions. For each fitted model, the operating threshold was selected by maximizing F1-score on the corresponding validation set and was fixed before test evaluation. Infested kernels were treated as the positive class. Balanced accuracy is the arithmetic mean of sensitivity and specificity. Standard deviations describe variation across partitions and training runs, not uncertainty across independent biological replicates. Conditional sampling intervals for sensitivity and specificity at the validation-selected thresholds are reported separately in Table S2b. Values were calculated using full-precision results and rounded to two decimal places for presentation.

Table S2b Sensitivity and specificity at validation-selected thresholds on a fixed test partition, with 95% conditional kernel-cluster bootstrap intervals

Nominal Moisture condition	Model	Sensitivity [95% interval]	Specificity [95% interval]
13%	1D-CNN	93.59 [90.38, 96.79]	88.12 [83.75, 92.50]
13%	Transformer	98.72 [96.79, 100.00]	10.62 [6.88, 14.37]
13%	CNN-Transformer	83.33 [77.56, 88.46]	95.00 [91.25, 98.12]
13%	HSI-MAE	96.79 [94.23, 98.72]	77.50 [70.62, 83.75]
13%	2D-CNN	89.74 [83.33, 95.51]	82.50 [72.50, 90.62]
13%	PLS-DA	91.67 [86.54, 96.15]	98.75 [96.88, 100.00]
13%	ADS-MAFNet	93.59 [90.38, 96.79]	86.88 [81.25, 91.88]
16%	1D-CNN	93.29 [89.63, 96.34]	40.00 [31.25, 48.75]
16%	Transformer	92.07 [85.98, 96.95]	8.12 [4.38, 12.50]
16%	CNN-Transformer	89.63 [85.37, 93.29]	45.00 [35.62, 54.37]
16%	HSI-MAE	88.41 [81.71, 93.90]	48.12 [36.88, 59.38]
16%	2D-CNN	85.98 [78.66, 92.68]	59.38 [47.50, 71.25]
16%	PLS-DA	93.90 [88.41, 98.78]	71.25 [61.25, 81.25]
16%	ADS-MAFNet	75.61 [68.90, 82.32]	75.62 [64.38, 85.62]
19%	1D-CNN	100.00 [100.00, 100.00]	0.00 [0.00, 0.00]
19%	Transformer	100.00 [100.00, 100.00]	0.00 [0.00, 0.00]
19%	CNN-Transformer	92.76 [89.47, 96.05]	11.25 [7.50, 15.00]
19%	HSI-MAE	100.00 [100.00, 100.00]	0.00 [0.00, 0.00]

Nominal Moisture condition	Model	Sensitivity [95% interval]	Specificity [95% interval]
19%	2D-CNN	85.53 [77.63, 92.11]	58.13 [48.12, 68.12]
19%	PLS-DA	90.79 [84.87, 95.39]	86.25 [79.38, 92.50]
19%	ADS-MAFNet	80.26 [73.68, 86.18]	73.75 [65.62, 81.88]
Mixed	1D-CNN	87.82 [85.26, 90.17]	43.54 [38.75, 48.33]
Mixed	Transformer	97.22 [95.73, 98.50]	7.92 [6.04, 9.79]
Mixed	CNN-Transformer	87.39 [84.62, 90.17]	48.12 [43.54, 52.71]
Mixed	HSI-MAE	91.45 [88.89, 94.02]	39.58 [34.79, 44.58]
Mixed	2D-CNN	83.97 [79.49, 88.25]	67.92 [62.29, 73.54]
Mixed	PLS-DA	92.09 [89.32, 94.66]	76.88 [72.08, 81.46]
Mixed	ADS-MAFNet	84.40 [80.98, 87.82]	69.38 [63.96, 74.79]

Note: Values are percentages expressed as point estimates [95% conditional bootstrap intervals], not ten-run means. The fixed test partition uses random seed 40, the first in the existing sequence 40-49. Fitted models and their previously selected validation thresholds were held fixed. Infested kernels were the positive class. Physical kernels were resampled with replacement within fixed moisture, class, and tray strata, retaining all four observations of each sampled kernel. Intervals are the 2.5th and 97.5th percentiles of 10,000 resamples; all resamples were valid. The test sets contained 79, 81, 78, and 237 physical kernels for 13%, 16%, 19%, and mixed moisture, respectively.

These intervals describe within-stratum sampling uncertainty conditional on the fitted models, locked thresholds, and observed acquisition structure. They do not include retraining, model or threshold selection, between-tray variation, or between-batch/container variation. Stratification within trays does not estimate tray effects. This retrospective internal analysis is not independent external validation. Degenerate intervals at 0% or 100% reflect the fixed predictions and observed sample, not certainty about performance in new samples or batches.

Table S3 Paired model differences on a fixed test partition

Differences are ADS-MAFNet minus the comparison model, in percentage points. The fitted models and classification rules match Tables 1 and 2.

Analysis	Nominal Moisture	Comparison model	Kernels	F1 difference [95% interval]	BA difference [95% interval]
Comparison	13%	1D-CNN	79	-1.41 [-4.77, 1.80]	-1.56 [-5.05, 1.88]
Comparison	13%	Transformer	79	24.02 [20.80, 27.11]	39.92 [36.45, 43.09]

Analysis	Nominal Moisture	Comparison model	Kernels	F1 difference [95% interval]	BA difference [95% interval]
Comparison	13%	CNN-Transformer	79	1.69 [-1.84, 5.39]	0.75 [-2.74, 4.23]
Comparison	13%	HSI-MAE	79	0.43 [-2.87, 3.72]	0.61 [-2.91, 4.09]
Comparison	13%	2D-CNN	79	3.33 [-1.26, 8.20]	3.19 [-1.55, 8.24]
Comparison	13%	PLS-DA	79	-4.07 [-8.67, 0.60]	-4.37 [-9.13, 0.36]
Comparison	16%	1D-CNN	81	1.80 [-3.58, 6.91]	9.94 [4.41, 15.49]
Comparison	16%	Transformer	81	8.50 [2.19, 14.41]	26.27 [20.08, 32.13]
Comparison	16%	CNN-Transformer	81	2.03 [-3.82, 7.51]	8.96 [2.45, 15.16]
Comparison	16%	HSI-MAE	81	2.08 [-3.36, 7.28]	7.66 [2.08, 13.28]
Comparison	16%	2D-CNN	81	-0.84 [-7.39, 5.45]	3.29 [-3.78, 10.40]
Comparison	16%	PLS-DA	81	-9.33 [-15.00, -3.79]	-7.93 [-13.85, -1.76]

Note: Neural models use the original two-logit argmax rule; PLS-DA uses a fixed score threshold of 0.5. The fixed partition uses seed 40, the first in the existing sequence 40-49. Each kernel contributes four observations. Intervals are the 2.5th and 97.5th percentiles of 10,000 paired kernel-cluster bootstrap resamples within fixed moisture, class, and tray strata. They are conditional on the fitted models and observed acquisition structure, not intervals for the ten-run means. They do not include retraining, selection, or between-tray/container uncertainty and are not multiplicity-adjusted. This is a retrospective internal analysis, not independent external validation. BA, balanced accuracy.

Table S3(continued) Paired model differences on a fixed test partition

Differences are ADS-MAFNet minus the comparison model, in percentage points. The fitted models and classification rules match Tables 1 and 2.

Analysis	Nominal Moisture	Comparison model	Kernels	F1 difference [95% interval]	BA difference [95% interval]
Comparison	19%	1D-CNN	78	11.26 [6.19, 16.13]	26.96 [21.88, 31.97]
Comparison	19%	Transformer	78	11.26 [6.19, 16.13]	26.96 [21.88, 31.97]
Comparison	19%	CNN-Transformer	78	11.87 [6.78, 16.74]	22.86 [17.20, 28.08]
Comparison	19%	HSI-MAE	78	11.26 [6.19, 16.13]	26.96 [21.88, 31.97]
Comparison	19%	2D-CNN	78	4.49 [0.19, 8.68]	9.14 [4.06, 14.16]
Comparison	19%	PLS-DA	78	-10.89 [-17.14, -4.93]	-10.89 [-16.97, -4.98]
Comparison	Mixed	1D-CNN	237	9.44 [6.42, 12.38]	6.90 [3.63, 10.07]
Comparison	Mixed	Transformer	237	12.46 [9.77, 15.16]	26.81 [23.58, 30.00]
Comparison	Mixed	CNN-Transformer	237	6.77 [3.61, 9.86]	6.18 [2.59, 9.64]
Comparison	Mixed	HSI-MAE	237	5.40 [2.57, 8.13]	7.11 [3.76, 10.38]
Comparison	Mixed	2D-CNN	237	1.14 [-1.67, 3.95]	0.77 [-2.29, 3.82]
Comparison	Mixed	PLS-DA	237	-6.58 [-9.82, -3.37]	-8.23 [-11.89, -4.58]
Ablation	Mixed	No-MAE	237	1.09 [-0.79, 2.94]	0.76 [-1.43, 2.87]
Ablation	Mixed	HSI-only	237	6.09 [3.48, 8.65]	9.56 [6.41, 12.74]
Ablation	Mixed	IMG-only	237	-0.55 [-3.22, 2.10]	-0.53 [-3.68, 2.53]

Note: Neural models use the original two-logit argmax rule; PLS-DA uses a fixed score threshold of 0.5. The fixed partition uses seed 40, the first in the existing sequence 40-49. Each kernel contributes four observations. Intervals are the 2.5th and 97.5th percentiles of 10,000 paired kernel-cluster bootstrap resamples within fixed moisture, class, and tray strata. They are conditional on the fitted models and observed acquisition structure, not intervals for the ten-run means. They do not include retraining, selection, or between-tray/container uncertainty and are not multiplicity-adjusted. This is a retrospective internal analysis, not independent external validation. BA, balanced accuracy.

Table S5 Training configurations and validation-based selection procedures for the Transformer analyses.

Item	Transformer in the main comparison	Transformer in the supplementary analysis
Input	Raw 256-dimensional mean spectrum	Same 256-dimensional mean spectrum with training-fitted bandwise standardization
Standardization fitting scope	Not applicable	Training subset of each partition; the same transformation applied to the validation and test subsets
Architecture	CLS-based Transformer	Same CLS-based architecture
Patch size / hidden dimension	16 / 128	16 / 128
Transformer layers / attention heads	2/4	2/4
Feedforward dimension / dropout rate	512 / 0.2	512 / 0.2
Pooling	CLS token	CLS token
Classification head	MLP: 128 → 128 → 64 → 2	Same
Optimizer / weight decay	AdamW / 1×10^{-4}	AdamW / 1×10^{-4}
Learning rate	Fixed at 3×10^{-4}	Candidates: 5×10^{-5} and 3×10^{-4}
Batch size	32	Up to 64
Maximum training epochs	100	60 per candidate
Early stopping	Patience of 20 epochs without a strict improvement in validation F1-score	Patience of 12 epochs without improvement under the checkpoint-selection criterion, subject to a minimum of 20 training epochs
Checkpoint selection	Highest validation F1-score; the earlier checkpoint retained in a tie	Highest validation F1-score; ties resolved by lower validation cross-entropy
Candidate configuration selection	No two-candidate search performed	Highest validation F1-score, followed by lowest validation cross-entropy; remaining ties resolved by the predefined candidate order
Classification rule for validation F1-score	Argmax over the two class logits	Infested-class softmax score > 0.5
Data partitions	Ten partitions per condition across four conditions; seeds 40–49	Same corresponding partitions
Supplementary search budget	Not applicable	40 partitions × 2 candidates = 80 candidate fits

Item	Transformer in the main comparison	Transformer in the supplementary analysis
Use of the test subset	Final evaluation	Final evaluation after configuration and checkpoint selection; not used for selection

Note: Standardization was fitted separately within each outer training subset. Candidate configurations and checkpoints were selected using the corresponding validation subset only. The supplementary analysis changed input scaling and several training settings; it was not a single-factor test of standardization. The 80 candidate fits correspond to 40 outer evaluations, not 80 independent data partitions.

Table S6 Test performance of the original and supplementary Transformer configurations across moisture conditions

S6A. Accuracy、Precision、Sensitivity and F1

Nominal Moisture condition	Configuration	Accuracy	Precision	Sensitivity	F1
13%	Original	52.18 ± 3.30	51.67 ± 2.28	98.59 ± 2.98	67.73 ± 1.26
13%	Supplementary	84.74 ± 2.05	84.63 ± 4.20	85.95 ± 3.85	85.15 ± 1.80
16%	Original	49.79 ± 1.09	49.79 ± 1.09	100.00 ± 0.00	66.47 ± 0.98
16%	Supplementary	70.81 ± 3.06	67.63 ± 3.46	80.01 ± 7.47	73.08 ± 3.58
19%	Original	48.19 ± 2.81	48.19 ± 2.81	100.00 ± 0.00	64.99 ± 2.60
19%	Supplementary	55.38 ± 9.65	54.07 ± 8.52	83.52 ± 12.36	64.48 ± 4.09
Mixed	Original	47.56 ± 3.29	47.56 ± 3.29	100.00 ± 0.00	64.40 ± 3.09
Mixed	Supplementary	71.52 ± 2.21	66.76 ± 3.09	79.76 ± 3.79	72.62 ± 2.62

S6B. Specificity、BA、AUC and Number of runs predicting all samples as positive

Nominal Moisture condition	Configuration	Specificity	BA	AUC	Number of runs predicting all samples as positive
13%	Original	4.25 ± 9.00	51.42 ± 3.03	0.557 ± 0.051	8/10
13%	Supplementary	83.50 ± 5.68	84.73 ± 2.06	0.928 ± 0.011	0/10
16%	Original	0.00 ± 0.00	50.00 ± 0.00	0.506 ± 0.066	10/10
16%	Supplementary	61.50 ± 8.91	70.75 ± 3.11	0.783 ± 0.035	0/10
19%	Original	0.00 ± 0.00	50.00 ± 0.00	0.498 ± 0.049	10/10
19%	Supplementary	29.88 ± 27.17	56.70 ± 8.02	0.602 ± 0.118	2/10
Mixed	Original	0.00 ± 0.00	50.00 ± 0.00	0.537 ± 0.030	10/10
Mixed	Supplementary	63.94 ± 4.07	71.85 ± 2.25	0.814 ± 0.021	0/10

Note: Accuracy, precision, sensitivity, specificity, balanced accuracy, and F1-score are percentages; AUC is reported on a 0–1 scale. Values are means ± sample standard deviations across ten partitions. "All-positive runs" denotes the number of runs in which every test record was classified as infested. Original and supplementary configurations used the corresponding classification rules specified in Table S5. These summaries describe variation across partitions and training runs, not independent biological replication.

Table S7 Architectural specifications of ADS-MAFNet and the comparison models

Model	Input and architecture
1D-CNN	Input: 1 × 256 mean spectrum. Conv1d(1, 64, kernel size 7, padding 3) → batch normalization → GELU → max pooling with size 2; Conv1d(64, 128, kernel size 5, padding 2) → batch normalization → GELU → max pooling with size 2; Conv1d(128, 128, kernel size 3, padding 1) → batch normalization → GELU → adaptive average pooling to length 1. The resulting 128-dimensional feature vector enters the shared classifier.
Transformer	The 256-dimensional spectrum is divided into 16 patches of 16 bands. Linear patch embedding maps 16 to 128 dimensions, followed by sinusoidal positional encoding and a CLS token, giving 17 tokens. Two pre-normalization Transformer encoder layers use an embedding dimension of 128, four attention heads, a feedforward dimension of 512, GELU activation, and dropout of 0.2. Layer normalization is applied to the CLS output, producing a 128-dimensional feature vector for the shared classifier.
CNN-Transformer	Parallel 1D-CNN and CLS-based Transformer pathways, as specified above. Their concatenated 256-dimensional features are passed through a Linear(256, 2) layer and softmax to obtain gating weights. The weighted concatenation, [g_c f_c; g_t f_t], has 256 dimensions and enters the shared classifier. This architecture differs from the serial spectral encoder used in ADS-MAFNet.
HSI-MAE	The pretrained serial spectral encoder specified below produces a 128-dimensional mean-pooled feature vector, which enters the shared classifier. Encoder dropout is 0.2 during supervised training. Encoder parameters are trainable from the start of supervised training.
2D-CNN	Input: 3 × 64 × 64 pseudocolor image. Three Conv2d blocks have channel dimensions 3

Model	Input and architecture
	→ 32 → 64 → 128. Each convolution uses a kernel size of 3, stride of 1, and padding of 1, followed by batch normalization and GELU. The first two blocks are followed by max pooling with size 2; the third is followed by adaptive average pooling to 1 × 1. Flattening → dropout of 0.2 → Linear(128, 128) → GELU produces a 128-dimensional feature vector for the shared classifier.
PLS-DA	Input: 256-dimensional mean spectrum. PLSRegression uses binary targets, with healthy = 0 and infested = 1. The candidate numbers of latent variables are 1, 2, 3, 5, 8, 10, 15, and 20. No neural classification head is used.
ADS-MAFNet	Inputs: 256-dimensional mean spectrum and 3 × 64 × 64 pseudocolor image. The serial spectral encoder and image encoder each produce 128-dimensional features. Direct concatenation gives a 256-dimensional vector. The classifier is LayerNorm(256) → Linear(256, 128) → GELU → dropout of 0.1 → Linear(128, 64) → GELU → dropout of 0.1 → Linear(64, 2). No gated-sum fusion is used.

Shared classifier for the five standalone neural comparators: LayerNorm(d) → Linear(d, 128) → GELU → dropout of 0.2 → Linear(128, 64) → GELU → dropout of 0.2 → Linear(64, 2), where d denotes the encoder feature dimension. All neural classifiers output two logits.

Serial spectral encoder used in ADS-MAFNet and HSI-MAE: The 256-dimensional spectrum is divided into 16 patches of 16 bands. Linear(16, 128) patch embedding and sinusoidal positional encoding are followed by a local CNN comprising two Conv1d(128, 128) layers with kernel size 3, padding 1, batch normalization, and GELU activation. A residual connection adds the local CNN output to the embedded tokens. Two pre-normalization Transformer encoder layers use an embedding dimension of 128, four attention heads, a feedforward dimension of 512, and GELU activation. Layer normalization is applied to the output tokens. Mean pooling over all 16 tokens produces a 128-dimensional feature vector; no CLS token is used in this encoder.

Abbreviations: BN, batch normalization; CLS, classification token; FFN, feedforward network; SNV, standard normal variate. Spectral inputs contain 256 bands. SNV is not applied.

Table S8 Training, model selection, data partitioning, and classification settings

S8A. Training and model-selection settings

Model or stage	Settings and selection procedure
Five standalone neural comparators	The comparators are 1D-CNN, Transformer, CNN-Transformer, HSI-MAE, and 2D-CNN. All use cross-entropy loss and AdamW with a learning rate of 3×10^{-4}, weight decay of 1×10^{-4}, batch size of 32, a maximum of 100 supervised epochs, and early-stopping patience of 20 epochs. No learning-rate scheduler is used. The checkpoint with the highest validation F1-score is retained; ties retain the earlier checkpoint. Test data are not used for checkpoint selection. Implementation: run_single_modal_by_moisture.py and run_single_modal_by_moisture_repeats.py.
ADS-MAFNet supervised training	Cross-entropy loss and AdamW are used with a learning rate of 5×10^{-5}, weight decay of 1×10^{-4}, batch size of 32, a maximum of 100 epochs, and early-stopping patience of 20 epochs. No learning-rate scheduler is used. The checkpoint with the highest validation F1-score is retained; ties retain the earlier checkpoint. Spectral-encoder parameter gradients are disabled during epochs 1–10 and enabled at epoch 11, when AdamW is reinitialized. Spectral-encoder and image-encoder dropout are 0.2; fusion-classifier dropout is 0.1. Freezing parameter gradients does not freeze batch-normalization running statistics.
MAE pretraining	The masking parameter is 0.45, giving floor(16 × 0.45) = 7 contiguous masked patches. Selected patch embeddings are replaced by a learned mask token, and all 16 token positions are encoded. The decoder is Linear(128, 128) → GELU → dropout of 0.1 → Linear(128, 16). MSE is calculated only for masked raw spectral values. Pretraining uses the current training subset only, AdamW with a learning rate of 1×10^{-3} and weight decay of 1×10^{-4}, batch size of 128, 100 epochs, and dropout of 0.1. The final pretrained encoder is transferred to supervised training; test reconstruction

Model or stage	Settings and selection procedure
	performance is not used to select the encoder. Implementation: pretrain_mae in run_single_modal_by_moisture.py and the CNNTransformerSpectralMAE model class.
PLS-DA	PLSRegression uses binary targets, scale=True, max_iter=1000, and tol=1e-6. Candidate numbers of latent variables are 1, 2, 3, 5, 8, 10, 15, and 20. Each candidate is fitted using the corresponding training subset only. No additional standardization is applied. Selection maximizes validation F1-score using a fixed score threshold of 0.5; ties favor fewer latent variables. Invalid dimensions, convergence failures, and nonfinite results are recorded. Predictions are continuous, uncalibrated, and unclipped scores. AUC is calculated from these scores; probability log-loss is not used for selection.
Supplementary Transformer diagnostic	The original Transformer architecture is retained. StandardScaler is fitted using training data only. Learning-rate candidates are 5×10^{-5} and 3×10^{-4}. AdamW uses weight decay of 1×10^{-4}. Training uses a maximum of 60 epochs, a minimum of 20 epochs, and early-stopping patience of 12 epochs. Batches are formed using array_split into ceil(n/64) batches, with a maximum batch size of 64. Candidate and checkpoint selection uses validation F1-score, followed by lower validation cross-entropy and then candidate order to resolve ties. Four conditions × ten partitions × two candidates give 80 candidate fits. These are not independent biological replicates. Implementation and diagnostics: train_additions.py.

Note: AdamW uses $\beta_1 = 0.9$, $\beta_2 = 0.999$, and $\varepsilon = 1 \times 10^{-8}$ for supervised neural training. The supplementary Transformer diagnostic is reported separately from the original Transformer comparison configuration. The PLS-DA search and neural-model procedures use different budgets and do not establish equivalent tuning effort.

S8B. Ablation settings

Ablation variant	Configuration and training differences
No-MAE	The fusion architecture is unchanged, but no MAE-pretrained weights are loaded. Supervised training starts from random initialization without initial spectral-encoder freezing. The learning rate is 5 × 10^{-5}.
HSI-only	The pretrained serial spectral encoder is followed by the shared MLP classifier with dropout of 0.2. No image encoder is used. Spectral-encoder parameters are frozen for the first ten supervised epochs and then fine-tuned. The learning rate is 5 × 10^{-5}.
IMG-only	The image encoder and classifier are the same as those used in the standalone 2D-CNN, with dropout of 0.2. No MAE pretraining is used. A dedicated run uses a learning rate of 5 × 10^{-5}.

Note: The ablations use the same supervised batch size, maximum epoch count, early-stopping patience, weight decay, and validation-F1 checkpoint-selection rule as ADS-MAFNet. HSI-only differs from the standalone HSI-MAE comparator in learning rate and initial encoder freezing. IMG-only differs from the standalone 2D-CNN comparator in learning rate. These results are not interchangeable, and their differences cannot be attributed solely to architecture.

S8C. Data partitioning, classification rules, and preprocessing

Item	Specification
Random seeds	The partition seeds are 40, 41, 42, 43, 44, 45, 46, 47, 48, and 49.
Physical kernel identity	Each physical kernel is identified by the combination of Moisture, Label, Source_Folder, and Grain_ID. All four sampling-time observations of each physical kernel remain in the same subset.
Partition allocation key	Class-stratified partitioning uses the coarser allocation key Label + local Grain_ID, targeting approximately 65%, 15%, and 20% training, validation, and test data, respectively. This allocation key is not a globally unique physical-kernel identifier. In single-moisture experiments, records are first restricted to the specified nominal moisture group.
Original classification rules	Neural models use the original two-logit argmax rule. PLS-DA uses a fixed score threshold of 0.5. Results from these rules are

Item	Specification
	reported separately from the validation-selected-threshold analysis.
Validation-selected thresholds	Thresholds maximize validation F1-score. Ties are resolved by higher specificity, then proximity to 0.5, and finally the larger threshold. One overall threshold is fixed for each fitted model and partition. Thresholds are not reselected using test moisture or sampling-time subgroups.
Repeated-run summaries	Results are summarized using ten-run means and sample standard deviations, with ddof=1. These summaries are descriptive because overlapping partitions are not independent biological replicates.
Original spectral neural inputs	The original spectral neural models use unstandardized 256-dimensional mean spectra. SNV is not applied.
Image inputs	Images are converted to channel-first floating-point arrays, and pixel values are divided by 255.
PLS-DA preprocessing	Internal centering and scaling are fitted using the corresponding training subset only.
Supplementary Transformer preprocessing	Bandwise standardization is fitted using the corresponding training subset only and applied to validation and test data.
MAE data restriction	MAE pretraining uses only the current training subset. Validation and test records are excluded from pretraining.
Implementation defaults	Source code specifies framework defaults, including normalization epsilon and activation implementation.
